# An mRNA Vaccine Based on Antigens From Conserved Regions of Monkeypox Virus A35R and M1R With a Dimer‐Like Conformation Confers Protection Against Both Monkeypox Virus and Vaccinia Virus Infections in Mice

**DOI:** 10.1002/mco2.70614

**Published:** 2026-01-22

**Authors:** Cong Tang, Longhai Yuan, Yun Xie, Yun Yang, Yanan Zhou, Junbing Wang, Hao Yang, Rui Peng, Jiali Xu, Wenhai Yu, Qing Huang, Wenqi Quan, Baisheng Li, Youchun Wang, Shuaiyao Lu

**Affiliations:** ^1^ Institute of Medical Biology Chinese Academy of Medical Sciences and Peking Union Medical College Kunming China; ^2^ Guangdong Provincial Center For Disease Control and Prevention Guangzhou China; ^3^ State Key Laboratory of Respiratory Health and Multimorbidity Beijing China; ^4^ Key Laboratory of Pathogen Infection Prevention and Control (Peking Union Medical College) Ministry of Education Beijing China; ^5^ Yunnan Key Laboratory of Cross‐Border Infectious Disease Control and Prevention and Novel Drug Development Kunming China; ^6^ Yunnan Provincial Key Laboratory of Vector‐borne Diseases Control and Research Kunming China

**Keywords:** mRNA vaccine, monkeypox virus (MPXV), vaccinia virus (VACV), vaccine antigen design

## Abstract

The 2022 global mpox outbreak caused by the monkeypox virus (MPXV) has underscored the urgent need for improved vaccine development. To address this need, we developed four candidate vaccine antigens based on conserved sequences of the MPXV A35R and M1R proteins utilizing a lipid nanoparticle (LNP) delivery system. All four vaccine candidates elicited varying degrees of humoral and cellular immune responses and conferred differential protection against MPXV and vaccinia virus (VACV) in BALB/c mice; notably, the dual‐antigen vaccines MV1 and MV2 induced more potent immunogenicity, including higher neutralizing antibody titers and cytokine secretion levels. However, among the four candidates, only the dual‐antigen vaccines MV1 and MV2 conferred protective efficacy in AGB6 mice and reduced infection‐induced pox lesion formation, indicating that antigens containing both intracellular mature virus (IMV) and extracellular enveloped virus (EEV) targets may be key to exerting robust protection. Notably, MV2—which was designed via structural truncation and recombination based on poxvirus‐broad‐spectrum antibodies using the AlphaFold3 prediction platform and adopts a single‐chain “dimer‐like” configuration—exhibited not only optimal protective efficacy but also sustained durable immune responses and protection. These findings indicate that MV2 induces favorable immunogenicity and has potential for preventing MPXV and VACV infections, supporting its promise as a clinical vaccine candidate for MPXV.

## Graphical abstract

We developed four candidate mRNA vaccine antigens based on conserved sequences of MPXV A35R and M1R proteins. Our findings demonstrate that the MV2 vaccine possesses favorable immunogenicity and prophylactic potential against MPXV and VACV infections, positioning it as a promising clinical candidate for MPXV prevention.

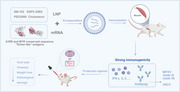

## 1 Introduction

The ongoing mpox outbreak is caused by monkeypox virus (MPXV), an enveloped double‐stranded DNA virus in the genus Orthopoxvirus of the family Poxviridae, which comprises two distinct clades: Clade I (with subclades Ia and Ib) and Clade II (with subclades IIa and IIb). First identified in Denmark in 1958, MPXV triggered a global mpox outbreak in 2022 [1]. Human mpox infection is typically mild, but severe disease and even fatalities can occur [2]. MPXV can enter the host via the oral or respiratory route, where it can infect mucosal surfaces of the oral cavity and respiratory tract, or can directly invade through broken skin. The virus subsequently replicates in keratinocytes, fibroblasts, and endothelial cells. The incubation period for MPXV infection ranges from 5 to 21 days. The vast majority of infected individuals develop skin lesions in the anogenital and trunk regions, while some patients also experience systemic symptoms such as headache and fever [3]. The World Health Organization (WHO) subsequently declared it a Public Health Emergency of International Concern (PHEIC) on two occasions (July 2022 and August 2024) [4]. As of February 2025, more than 100 countries and regions have reported 127,905 confirmed cases of mpox, with 283 associated fatalities.

Vaccines represent among the most critical public health interventions in human history, and the administration of safe and effective vaccines remains the most efficient strategy for controlling infectious disease transmission. Currently, only three smallpox live‐attenuated vaccines (JYNNEOS, LC16m8, and ACAM2000) are approved for MPXV prophylaxis [5]. There is a pressing need for more mpox vaccines utilizing novel vaccine platforms to be deployed.

The mRNA vaccine platform represents a novel immunization strategy in which the core component is messenger ribonucleic acid (mRNA). This platform operates by delivering mRNA encoding specific antigenic proteins into the body through lipid nanoparticles (LNPs) or other delivery systems, and antigen‐presenting cells (APCs) process these external antigens through the endogenous pathway, thereby activating both T cells and B cells [6]. In addition, immune responses are subsequently induced [7, 8]. Characterized by high immunogenicity and rapid development capabilities, mRNA vaccines have made significant contributions to pandemic containment during the COVID‐19 outbreak [9].

MPXV exists in two distinct infectious forms: an intracellular mature virus (IMV) and an extracellular enveloped virus (EEV) [10, 11]. In MPXV vaccine research, IMV antigens, including A21, A29, M1R, and H3, along with EEV antigens, such as B6R and A35R, have been demonstrated to induce immunogenic responses [12, 13, 14, 15, 16]. Current mRNA vaccine development for MPXV predominantly focuses on multiantigen cocktail approaches, which have shown moderate protective effects [17]. However, such mRNA vaccine mixtures not only significantly increase production complexity and costs but also may lead to issues such as antigenic competition, potentially compromising their immunogenic efficacy. Notably, compared with these combination strategies, single‐antigen formulations exhibit relatively poor protective efficacy.

Based on the conserved sequences of A35R and M1R, in this study, we designed four vaccine antigens delivered using a LNP formulation. All the candidate vaccines elicited significant humoral and cellular immune responses in BALB/c mice. Subsequent challenge experiments with MPXV and vaccinia virus (VACV) demonstrated varying degrees of protection across the vaccine groups. To further evaluate the efficacy of the vaccine against MPXV infection, immunogenicity and protection were assessed in interferon signaling‐deficient AGB6 mice, which developed the typical clinical symptoms of pox lesions upon MPXV infection. Among the candidates, the MV2 vaccine exhibited the best overall protective efficacy. Long‐term immunogenicity evaluation of the MV2 vaccine revealed that immunized mice maintained considerable immune responses up to 280 days after the initial immunization and that the vaccine still conferred certain protection against MPXV in BALB/c mice, supporting its good durability. These results demonstrated the feasibility of the structurally truncated and reconstituted single‐chain “dimer‐like” antigen design. The MV2 mRNA vaccine developed using this strategy exhibits promising immunogenicity and protective efficacy, highlighting its potential as a clinical candidate for curbing the global spread of MPXV.

## 2 Results

### 2.1 In Vitro Expression of Designed Immunogens and Characterization of mRNA‐LNPs

**FIGURE 1:**
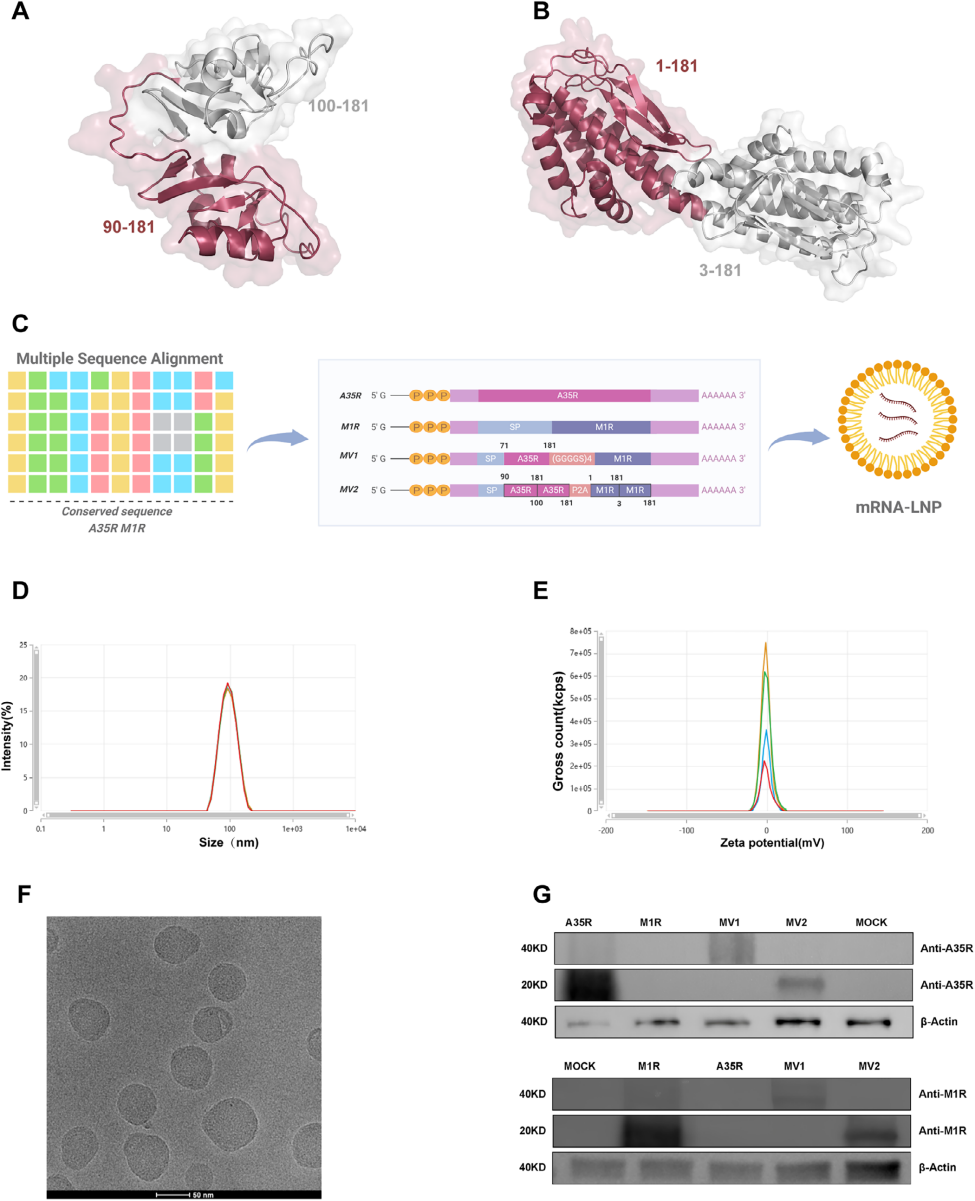
In vitro expression of designed immunogens and characterization of mRNA‐LNPs. (A, B) A35R/M1 “dimer‐like” structure prediction. Through computational structural modeling employing AlphaFold3, we predicted the “dimer‐like” conformational states of the target viral proteins A35R (A) and M1R (B). (C) Vaccine design. Utilizing bioinformatics methods, we performed multiple sequence alignment (MSA) on the amino acid sequences of the MPXV A35R and M1R proteins retrieved from the NCBI database. Following the identification of conserved regions through this comparative analysis, four distinct vaccine candidates were rationally designed based on these evolutionarily preserved epitopes (created with BioRender.com). (D, E) Physicochemical characterization of mRNA‐LNP nanoparticle formulations was performed through hydrodynamic diameter and zeta potential analyses. Through physicochemical characterization of synthesized mRNA‐LNP nanoparticle formulations, the hydrodynamic diameter (D) and surface charge (zeta potential) (E) were analyzed. The system exhibited an approximately 90 nm particle size with a monodispersed zeta potential profile. The measured parameters, including the polydispersity index (PDI < 0.1) and quality factor (>1), demonstrated a narrow size distribution and optimal colloidal stability. F) Cryo‐electron microscopy (Cryo‐EM) images. (G) Western blot images.

We employed AlphaFold3 to predict the antigen‒antibody docking between the MPXV clade II strains A35R and M1R and their respective neutralizing antibodies (A27D7/7D11). The results revealed that the interactions were predominantly localized to the head region (Figure S1A,B), which aligned with prior reports [12]. To increase effective epitope exposure, head fragments of A35R and M1R were generated via selective removal of the stalk region. Subsequently, “dimer‐like” conformational antigens were created by repeating these head fragments. AlphaFold3 was employed to model the “dimer‐like” conformation and predict antigen‒antibody docking with the neutralizing antibody A27D7/7D11. Compared with the untreated A35R and M1R, the “dimer‐like” conformational antigens exhibited more binding sites with their corresponding antibodies, thereby enhancing the antigen‒antibody interactions to some extent (Figure S1C,D). To increase the breadth of vaccine coverage, we adopted the strategy of utilizing conserved sequences, which yielded favorable results in our previous study [18]. A total of 7258 A35R and 7011 M1R amino acid sequences derived from various MPXV strains were retrieved from the NCBI database, after which multiple sequence alignment was performed to identify conserved regions for both antigens. In accordance with our previous method, we designed a “dimer‐like” conformation using the conserved sequences of A35R and M1R (Figure 1A,B, Figure S4). Subsequent antigen‒antibody docking prediction analysis with reported neutralizing antibodies against orthopoxviruses indicated that interactions were observed in all the cases, with predictions demonstrating a certain level of confidence (Figures S2 and S3) [19, 20, 21, 22, 23]. Based on these conserved sequences, four mRNA vaccine candidates were designed (Figure 1C). The A35R and M1R vaccines encoded single antigens, whereas the MV1 vaccine incorporated a soluble A35R fragment connected to full‐length M1R via a (GGGGS)4 linker peptide. P2A is a self‐cleaving peptide linker [24]. We utilized P2A to connect the two “dimer‐like” antigens, generating MV2, which ensured the structural stability of both antigens and enabled single‐chain delivery of dual antigens.

Plasmids were linearized and transcribed into RNA, the purity of which was preliminarily assessed by denaturing gel electrophoresis. LNPs were formulated by mixing the cationic lipids SM‐102, PEG2000‐DMG, DSPC, and cholesterol at a ratio of 50:1.5:10:38.5, followed by microfluidic assembly to produce mRNA‐LNPs. Characterization revealed that the mRNA‐LNPs exhibited uniform particle sizes of approximately 90 nm (PDI < 0.1) (Figure 1D), monodispersed zeta potential profiles (quality factor > 1) (Figure 1E), and encapsulation efficiencies exceeding 90%, indicating high homogeneity and encapsulation efficacy. In addition, we investigated the dsRNA content generated during mRNA production and the stability of the LNPs. The results revealed low levels of dsRNA in the produced mRNA, and the particle size and encapsulation efficiency of the formulated LNPs remained stable and did not significantly change over 96 h, further demonstrating their favorable stability profile (Figure S5). Cryo‐electron microscopy (cryo‐EM) further confirmed the LNP morphology (Figure 1F). Finally, transfection of 293T cells with mRNA‐LNPs resulted in successful antigen expression, as determined by western blot analysis (Figure 1G, Figure S6).

### 2.2 The Vaccine Group Demonstrates Rapid Activation of the Immune System and Elicits Robust Humoral Immune Responses

**FIGURE 2:**
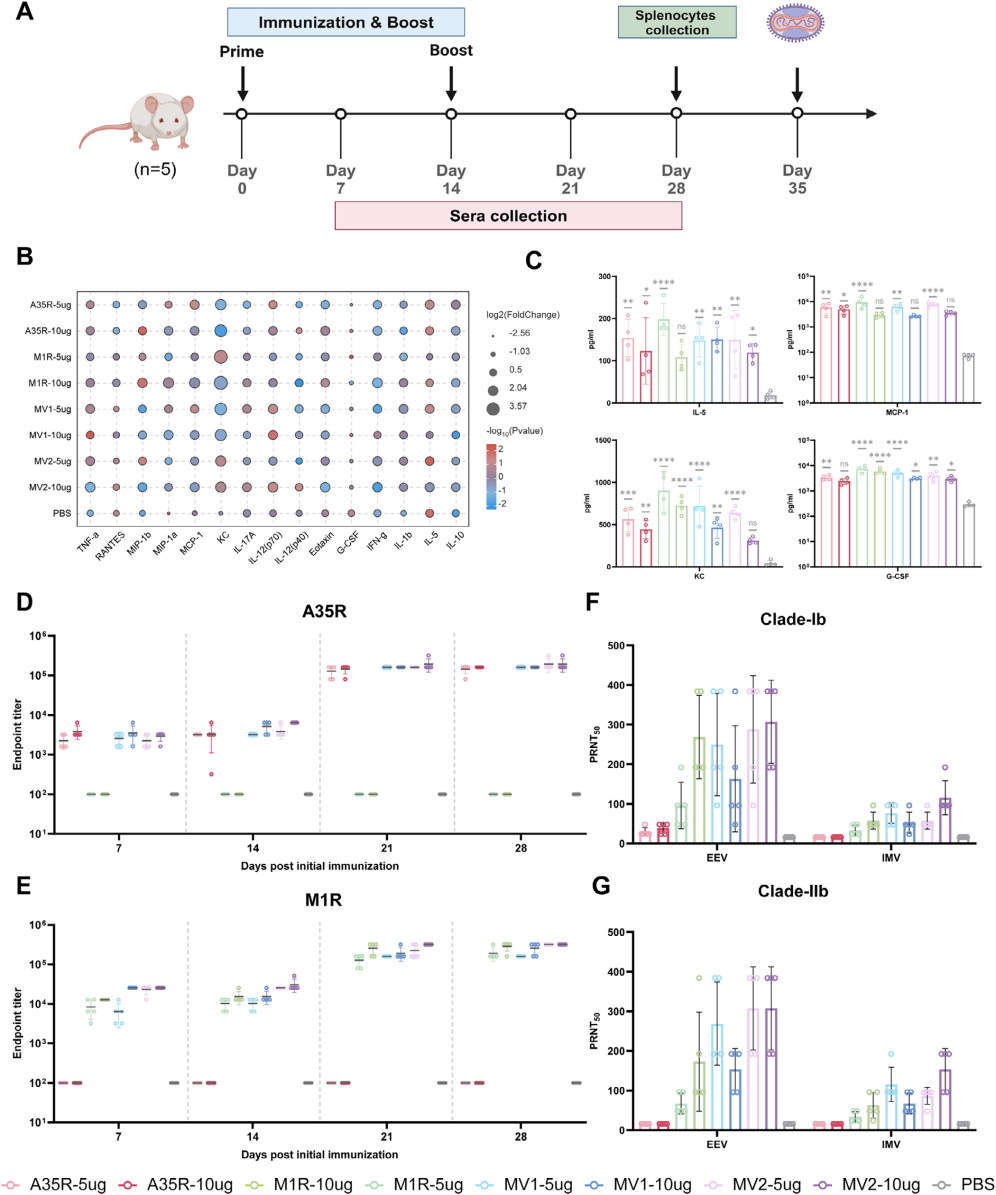
The vaccine group demonstrates rapid activation of the immune system and elicits robust humoral immune responses. (A) Vaccination and sample collection timeline (created with BioRender.com). (B) Changes in relevant cytokine levels at 5 and 24 h post‐immunization, logarithmically transformed; log_2_FC indicates the ratio of cytokine levels at 5–24 h. (C) Quantitative profiling of serum immunomodulatory cytokine secretion dynamics was conducted at 5 h post‐immunization. (D, E) Quantitative measurement of antigen‐specific binding antibody titers against the viral protein A35R or M1R in murine serum was performed via enzyme‐linked immunosorbent assay (ELISA). (F, G) Neutralizing antibody titers specific to Mpox virus (MPXV clade Ib and IIb) in murine serum were quantitatively determined using a live virus neutralization assay. Statistical analysis was conducted using one‐way ANOVA and Tukey's multiple comparison test for bar graphs: *p < 0.05; **p < 0.01; ***p < 0.001; ****p < 0.0001; ns, not significant.

To evaluate the immunogenicity of the mRNA vaccines, we implemented a prime‐boost immunization strategy with 14‐day intervals and administered high‐dose (10 µg) and low‐dose (5 µg) intramuscular injections to healthy female BALB/c mice on days 0 and 14. Serum samples were collected at 5 h, 24 h, 7 days, 14 days, 21 days, and 28 days after primary immunization (Figure 2A).

First, we analyzed serum cytokine levels at 5 and 24 h postimmunization to assess immediate immune responses. The analytical results revealed elevated levels of multiple cytokines at 5 h compared with 24 h postimmunization in the vaccinated group (Figure 2B). Furthermore, compared with the PBS control group, the vaccinated group exhibited increased levels of immunomodulatory cytokines, including IL‐5, KC, MCP‐1, and G‐CSF, at 5 h postimmunization (Figure 2C). IL‐5 plays a critical role in immune regulation by stimulating B‐cell proliferation, differentiation, and antibody production, thereby enhancing humoral immunity [25, 26]. As a chemokine, KCs facilitate immune cell mobilization and promote the directional migration of specific leukocyte populations [27]. MCP‐1, a pivotal chemokine, binds to its receptor CCR2 to activate monocytes and other immune cells, directing leukocyte infiltration and modulating T‐cell proliferation and functionality [28, 29, 30, 31]. G‐CSF, a pleiotropic cytokine, regulates neutrophil maturation and migration while modulating monocyte and macrophage activity [32, 33, 34]. The observed dynamic changes in these immunomodulatory cytokines indicated rapid activation of the immune system in the vaccinated group.

Subsequent evaluation of A35R‐ and M1R‐specific binding antibody levels at 7, 14, 21, and 28 days post‐immunization revealed robust antibody induction by all four vaccine candidates (Figure 2D,E). Notably, the elicited binding antibody titers exhibited no dose‐dependent relationship. On Day 28 after the primary immunization, all the vaccine groups elicited modest neutralizing antibody titers against MPXV clade Ib or clade IIb, including those directed against EEV and IMV (Figure 2F,G). Collectively, these results demonstrated rapid immune system activation and effective humoral immune responses in vaccinated mice.

### 2.3 The Vaccine Group Exhibited Robust Cellular Immune Responses

**FIGURE 3:**
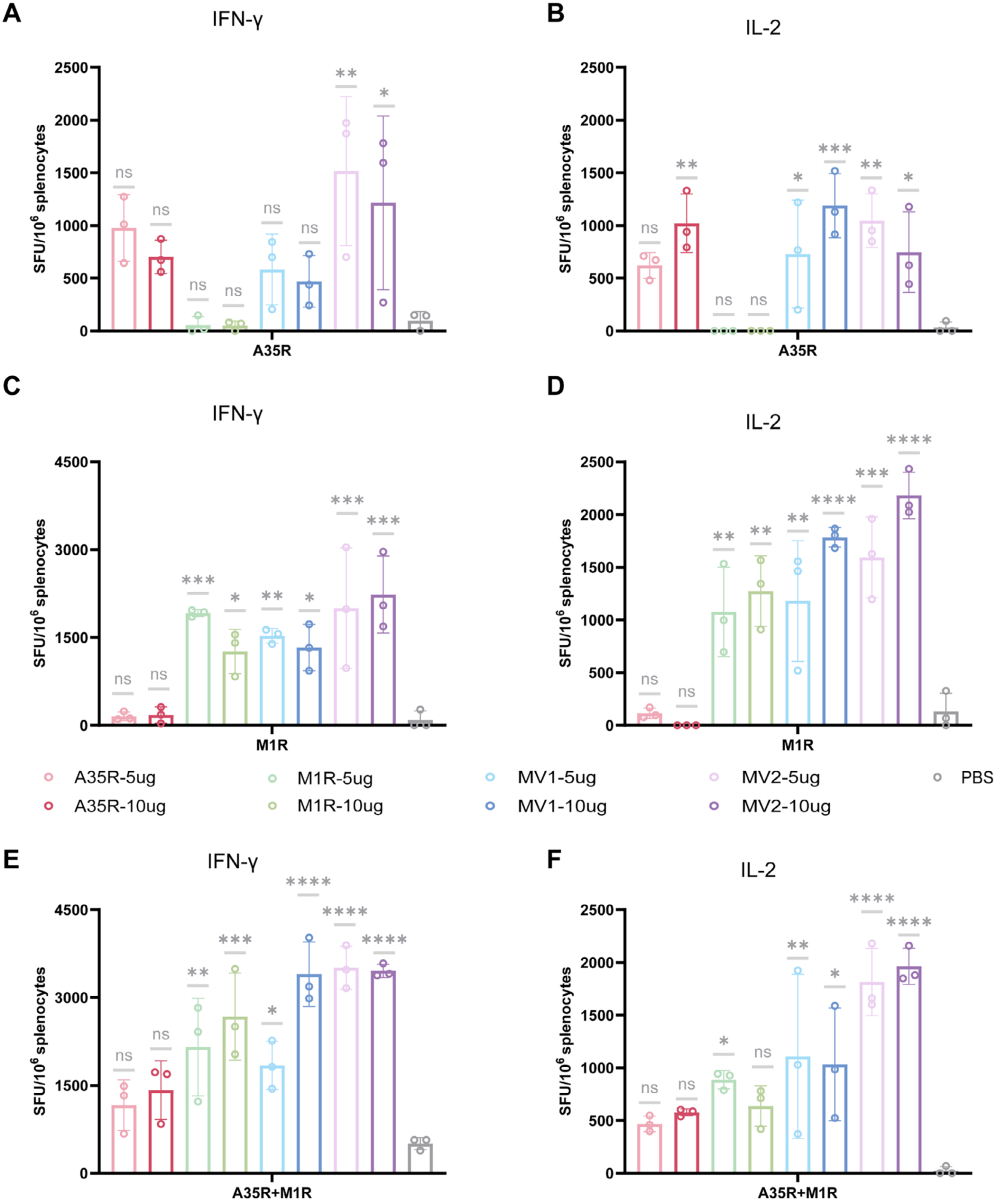
The vaccine group exhibited robust cellular immune responses. (A) Detection of IFN‐γ secretion levels using A35R protein as a stimulus. (B) Detection of IL‐2 secretion levels using A35R protein as a stimulus. (C) Detection of IFN‐γ secretion levels using M1R proteins as stimuli. (D) Detection of IL‐2 secretion levels using M1R protein as a stimulus. (E) Detection of IFN‐γ secretion levels using A35R and M1R protein as a stimulus. (F) Detection of IL‐2 secretion levels using A35R and M1R proteins as a stimulus. Statistical analysis was conducted using one‐way ANOVA and Tukey's multiple comparison test for bar graphs: *p < 0.05; **p < 0.01; ***p < 0.001; ****p < 0.0001; ns, not significant.

To evaluate the T‐cell‐mediated immune responses induced by the vaccines, splenocytes were harvested from immunized mice at 35 days post‐vaccination and subjected to Elispot assays using A35R (Figure 3A,B), M1R (Figure 3C,D), or M1R+A35R (Figure 3E,F) combined proteins as stimulants for IFN‐γ (Figure S7) and IL‐2 (Figure S8) detection [35]. The results revealed elevated IFN‐γ and IL‐2 cytokine levels across all the vaccine groups. Interestingly, we observed that compared with A35R stimulation, M1R stimulation triggered substantially higher cytokine levels in the MV1‐ and MV2‐immunized groups, suggesting that the M1R antigen component might exhibit superior cell‐mediated immunogenicity within these dual‐antigen vaccine constructs.

### 2.4 The mRNA Vaccines Confer Varying Degrees of Protection Against MPXV in BALB/c Mice

**FIGURE 4:**
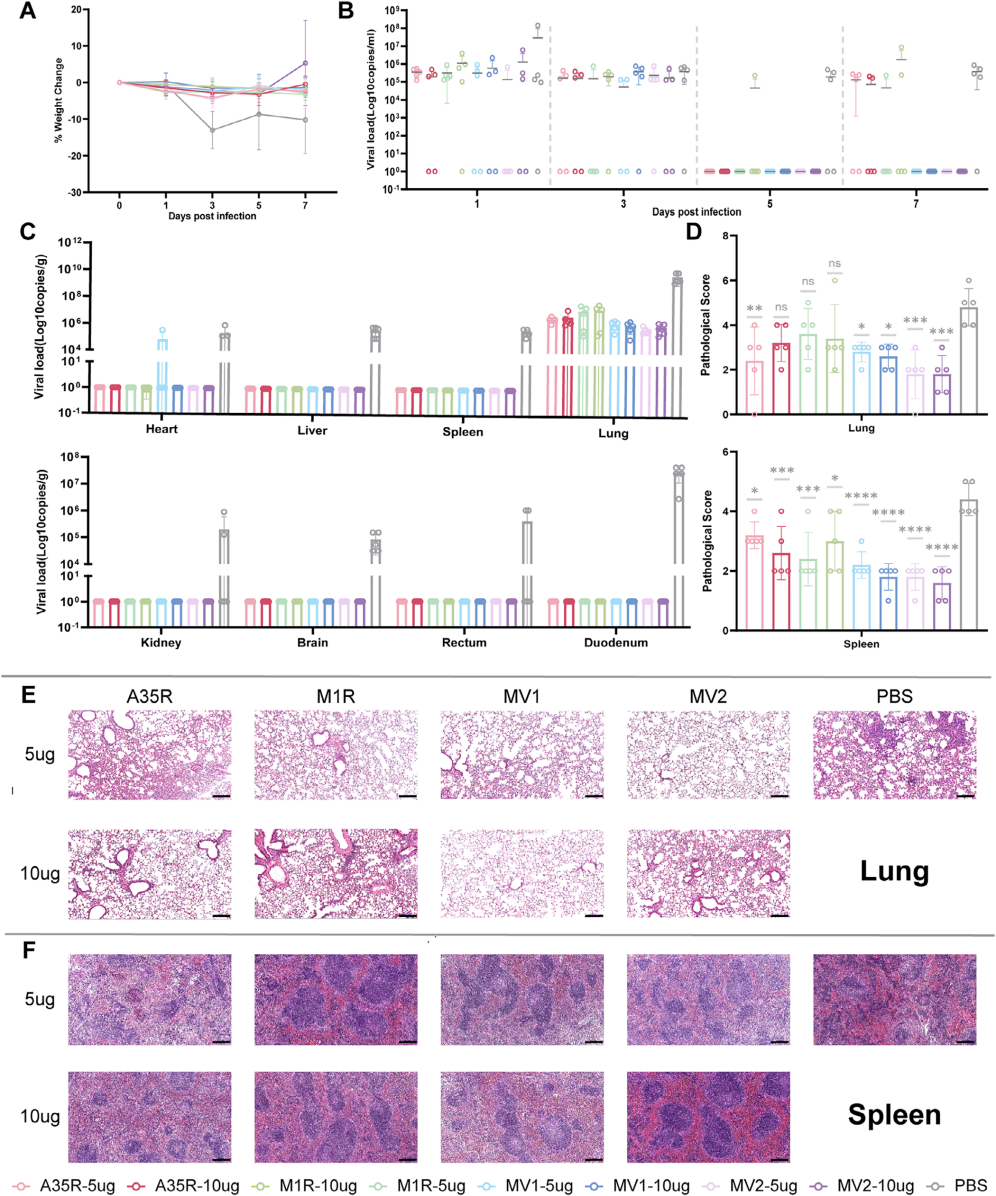
The mRNA vaccines confer varying degrees of protection against MPXV in BALB/c mice. (A) Schematic representation of longitudinal body weight monitoring in murine subjects following pathogen challenge. (B) Quantitative analysis of viremia kinetics in murine models following experimental viral challenge. (C) Quantitative analysis of viral load distribution across multiple organ systems in mice was conducted at 7 days post‐inoculation. (D) Lung and kidney pathological score. (E, F) Lung and kidney pathological damage and pathological scoring caused by MPXV infection in BALB/c mice.

To evaluate the protective efficacy of the vaccines against MPXV, we conducted a viral challenge experiment. The mice were intranasally challenged with 1 × 10^6^ PFU of MPXV 28 days post‐immunization. Body temperature (Figure S9) and weight were monitored throughout the study period, while viral loads in throat swabs and blood samples were measured at various time points. The mice in the PBS control group exhibited significant weight loss post‐challenge, whereas all the mice in the vaccine groups maintained stable body weights with only minor fluctuations (Figure 4A). Notably, complete viral clearance in the bloodstream was achieved by Day 5 post‐challenge in the MV1 and MV2 vaccinated groups, whereas substantial MPXV viremia persisted until Day 7 in the PBS‐treated mice (Figure 4B).

At the experimental endpoint (Day 7 post‐challenge), the mice were euthanized for comprehensive viral load assessment across multiple tissues. With the exception of pulmonary samples, no detectable viral loads were detected in any organs from the vaccinated groups, in contrast to the consistently high viral loads in the PBS controls. The observed viral persistence in lung tissues across all groups likely reflected the intranasal challenge route, although compared with the PBS control groups, the vaccinated groups demonstrated variably reduced pulmonary viral loads. Notably, compared with the other vaccine groups, the MV2‐immunized group presented significantly lower pulmonary viral loads (Figure 4C). To comprehensively evaluate the protective efficacy of the vaccine, we conducted pathological analysis of multiple organs collected from challenged mice at 7 days post‐infection. The results revealed varying reductions in pathological damage in the vaccinated groups across multiple tissues/organs (Figure 4D, Figure S11), particularly in the kidney (Figure S11A), lung (Figure 4E), and spleen (Figure 4F). Notably, both the high‐ and low‐dose MV2 groups showed a complete absence of renal hemorrhage and a significant reduction in inflammatory cell infiltration in the kidney, while the other vaccines also exhibited certain renoprotective effects. Owing to the intranasal challenge method, the lungs exhibited high viral loads, leading to severe pathological manifestations, including alveolar/interstitial hemorrhage, thrombosis, and inflammatory infiltration. Neither the A35R nor the M1R vaccine provided effective pulmonary protection, as determined by histopathological assessment, whereas high‐dose MV2 treatment significantly mitigated lung damage, indicating superior protective efficacy. As the primary immune regulatory organ, the spleen plays crucial roles in antiviral responses. MPXV‐induced splenic damage manifested as lymphocyte infiltration and diffuse hemorrhage, both of which were significantly alleviated by the MV1 and MV2 vaccines. Among the other examined tissues, the MV2 vaccine reduced organ damage to varying degrees (Figures S10 and S11). Collectively, these pathological findings indicated that all four vaccine candidates differentially reduced tissue damage, with the MV2 vaccine providing optimal protection—a conclusion consistent with the viral load measurements.

### 2.5 The mRNA Vaccines Protect Mice From Lethal‐Dose VACV Challenge

**FIGURE 5:**
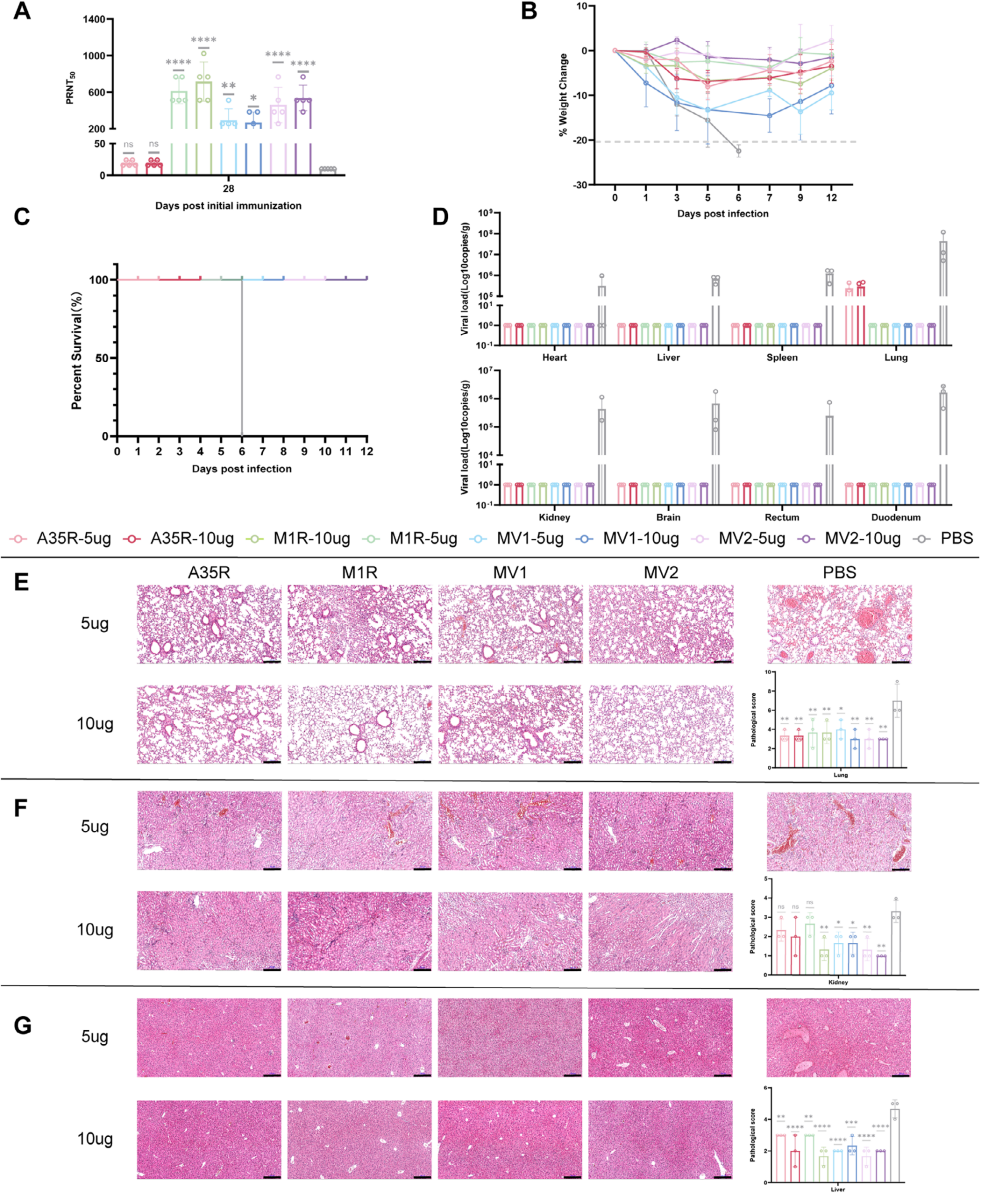
mRNA vaccines protect mice from lethal‐dose VACV challenge. (A) Neutralizing antibody titers against VACV elicited by vaccine immunization in mice. (B) Schematic representation of longitudinal body weight monitoring in murine subjects following pathogen challenge. (C) Survival curve of each group after challenge with the virus. (D) Quantitative analysis of viral load distribution across multiple organ systems in mice. (E) Lung pathological damage and score. (F) Kidney pathological damage and score. (G) Liver pathological damage and score. Statistical analysis was conducted using one‐way ANOVA and Tukey's multiple comparison test for bar graphs: *p < 0.05; **p < 0.01; ***p < 0.001; ****p < 0.0001; ns, not significant.

Since our vaccine design was based on broad‐spectrum orthopoxvirus antibodies, we measured the neutralizing antibody titers against VACV elicited by the vaccines. The results indicated that all four vaccine candidates elicited detectable levels of VACV‐neutralizing antibodies (Figure 5A). To further investigate the protective efficacy of mRNA vaccines against orthopoxviruses, we challenged immunized mice with 30 LD50 of VACV‐VTT. PBS‐treated mice exhibited significant weight loss and succumbed to infection by Day 6 post‐challenge, whereas vaccinated mice showed only mild weight loss, and all the mice survived until Day 12 (Figure 5B,C). To comprehensively assess vaccine‐mediated protection, we performed necropsies on the mice in the PBS group on Day 6 and the vaccinated groups on Day 12 post infection. We then collected multiple organs for viral load quantification and histopathological evaluation.

Viral load analysis revealed undetectable viral titers in all the vaccine groups except for A35R, which resulted in a measurable viral load in lung tissue. In contrast, the viral loads in all the organs examined were high in the PBS‐treated mice (Figure 5D). Pathological analysis of various organs revealed substantially more severe tissue damage in the PBS control group than in the vaccinated group, particularly in the lung (Figure 5E), kidney (Figure 5F), and liver (Figure 5G) tissues.

The lungs exhibited the most pronounced pathology, characterized by alveolar hemorrhage, interstitial inflammation, and cellular infiltration. All the vaccine formulations mitigated these pathological manifestations to varying degrees. In the kidneys, the hemorrhagic lesions and inflammatory cell infiltration observed in the PBS controls were markedly reduced in the vaccinated groups. Notably, only one mouse in the MV2 vaccine group displayed hemorrhage, indicating superior renal protection. Hepatic pathology revealed vascular congestion, hemorrhage, and lymphocytic infiltration, which were significantly alleviated by vaccination. Compared with the PBS control group, the vaccinated group also exhibited reduced pathological damage to other organs (Figures S12 and S13).

These findings demonstrated that, while all the vaccine candidates conferred varying levels of protection against VACV challenge, MV2 exhibited the most robust protective efficacy, particularly in preserving renal and hepatic integrity, which was consistent with its ability to reduce the viral load.

### 2.6 MV1 and MV2 Confer Effective Protection in AGB6 Mice and Significantly Reduce Pox Lesion Formation

**FIGURE 6:**
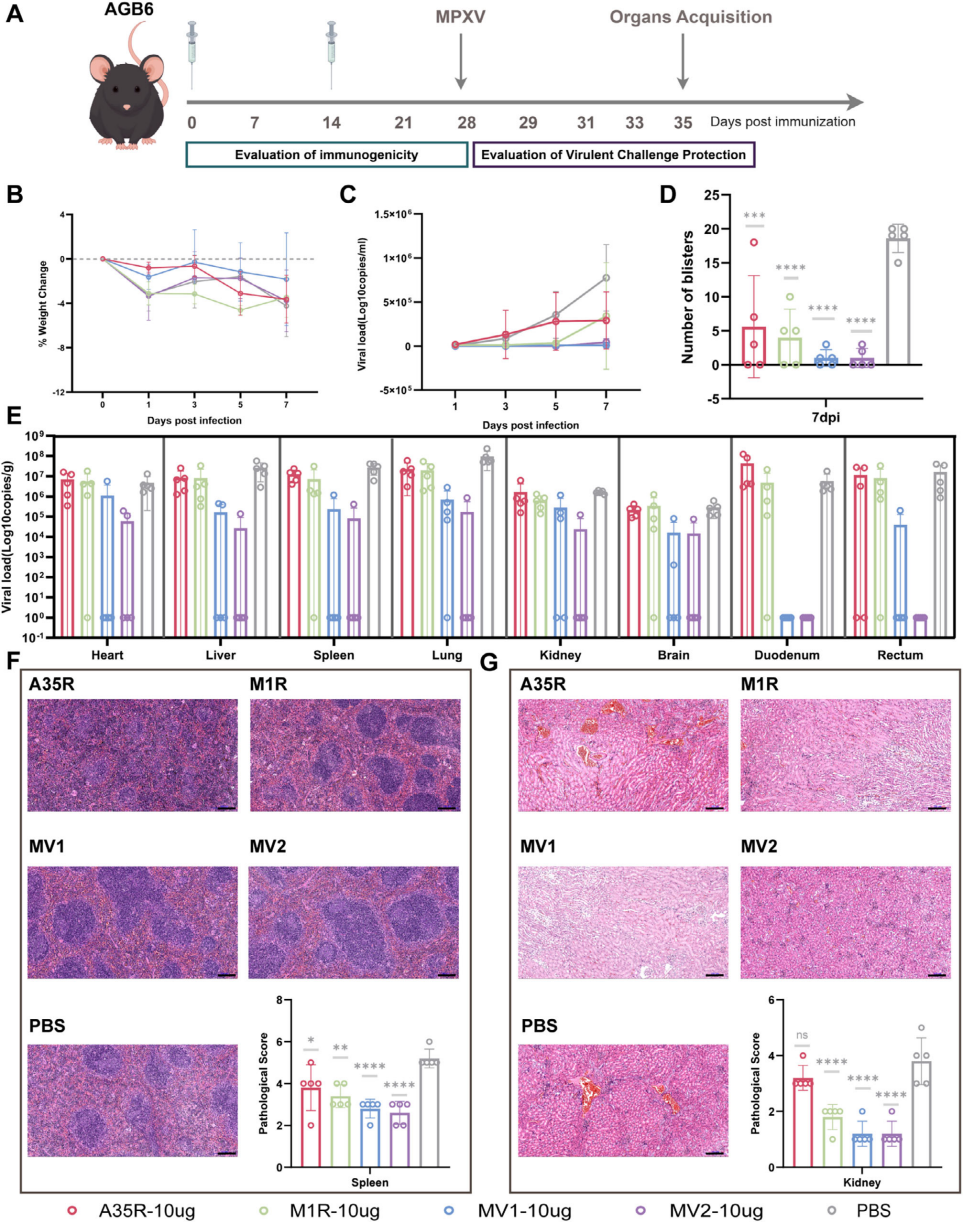
MV1 and MV2 confer effective protection in AGB6 mice and significantly reduce pox lesion formation. (A) Schematic diagram of the immunogenicity and challenge protection evaluation in AGB6 mice. (B) Changes in body weight following MPXV infection. (C) Viral replication levels in mouse blood during the infection course. (D) Number of pox lesions in mice on Day 7 post infection. (E) Viral replication levels in organs collected from mice sacrificed on Day 7. (F) Pathological injury and histopathological scores of the spleen of vaccinated and control AGB6 mice after infection. (G) Pathological injury and histopathological scores of the kidney of vaccinated and control AGB6 mice after infection.

Interferon signaling‐deficient AGB6 mice develop pox lesions following MPXV infection [36]. To further evaluate the protective efficacy of the four candidate vaccines and their control over typical clinical manifestations, AGB6 mice were immunized with 10 µg of each vaccine following the same immunization and challenge protocol as that used for BALB/c mice (Figure 6A). All four vaccines elicited strong humoral and cellular immune responses in AGB6 mice, albeit these responses were weaker than those observed in BALB/c mice (Figure S14). Following intranasal challenge with MPXV at the same dose, no significant changes in body weight were observed in either the vaccine group or the control group of mice (Figure 6B), and viral loads were detected in the blood and multiple organs of infected AGB6 mice, accompanied by severe pathological damage (Figure S15), particularly in the spleen and kidneys. The bivalent antigen vaccines MV1 and MV2 significantly reduced the viral load in the blood. Notably, in the MV2 group, detectable viremia was delayed until Day 7 post‐challenge (Figure 6C). Although the A35R and M1R monovalent vaccine groups showed some control over viremia, they did not effectively reduce the viral load in tissues or mitigate pathological injury. In contrast, MV1 and MV2 demonstrated superior protection, significantly suppressing MPXV replication across all the examined organs (Figure 6E) and markedly reducing the extent of pathological damage, especially in the spleen and kidneys (Figure 6F,G). Notably, MV2 exhibited the best overall protective profile: Among all five mice in the MV2 group, only one showed detectable viral replication in the lung and spleen, and no virus was detected in the duodenum or rectum. All the mice in the A35R, M1R, and control groups developed pox lesions on the tail by Day 7. Some mice in the MV1 and MV2 groups also developed lesions; however, compared with the control treatment, both MV1 and MV2 significantly reduced the incidence of lesions. Moreover, only in the MV1 and MV2 groups did some mice remain completely free of lesions, and the number of lesions was significantly lower in the MV1 and MV2 groups than in the other groups (Figure 6D). These results indicated that the monovalent mRNA vaccines A35R and M1R, targeting a single viral form, did not provide sufficient protection in AGB6 mice. In contrast, the bivalent mRNA vaccines MV1 and MV2, delivering two antigenic targets, not only significantly suppressed viral replication but also partially controlled pox lesion formation. The single‐chain “dimer‐like” antigen vaccine MV2 demonstrated the most comprehensive protective efficacy.

## 3 Discussion

Since 2022, the global mpox outbreak has persisted with continuous development, necessitating the development of safe and effective vaccines. The currently approved MPXV vaccines are exclusively live‐attenuated smallpox vaccines, which have limitations including suboptimal immunogenicity and safety concerns. The mRNA vaccine platform, which has strong immunogenicity and rapid production capabilities, has proven exceptionally effective during the COVID‐19 pandemic and has made substantial contributions to epidemic control [9].

Capitalizing on the dual virion formation characteristics of MPXV (EEV and IMV), we selected the immunodominant antigens A35R and M1R as vaccine targets. Using antigenic epitopes of broad‐spectrum antibodies and multiple sequence alignment, we identified conserved sequences as the foundation for the development of four vaccine candidates. The A35R and M1R vaccines encode single targets, whereas MV1 and MV2 use single‐chain delivery systems to coexpress both targets. We engineered repeat sequences of head fragments from A35R and M1R to create dimer‐like configurations. To ensure the structural stability of the dual targets, the MV2 vaccine incorporates a cleavable linker peptide (P2A) to connect these dimer‐like sequences, achieving coordinated delivery of both targets through a single‐chain architecture.

Current mpox vaccine development strategies predominantly employ cocktail approaches involving three or more antigens in tandem or mixed formulations, which have demonstrated favorable efficacy, and current multiantigen single‐chain delivery approaches for MPXV vaccines primarily rely on simple tandem linkage of sequences [37, 38, 39, 40]. In our study, based on broad‐spectrum antibody epitopes of orthopoxviruses, we developed MV2—a “single‐chain pseudodimer” vaccine engineered through structural modification of antigens derived from different viral forms of MPXV. With only two antigens, MV2 achieved levels of immunogenicity and protective efficacy comparable to those reported for existing MPXV vaccine candidates while also demonstrating cross‐protective efficacy against orthopoxviruses. Notably, monovalent antigen configurations yielded suboptimal protective outcomes, as evidenced by challenge protection data (Figures 4, 5, 6). These findings suggest that bivalent antigen formulations may be sufficient to confer adequate protection against MPXV, whereas single‐antigen vaccines may be insufficient for achieving robust prophylactic effects.

We conducted a comprehensive evaluation of the immunogenicity profiles across four vaccine candidates, encompassing both humoral and cellular immunity. All four candidate vaccines exhibited robust immunogenicity and elicited effective neutralizing antibodies against both clade I and clade II MPXVs, indicating a certain range of protection against MPXV. This effect might be attributable to the conserved sequence strategy. Notably, the MV2 vaccine demonstrated superior performance in eliciting both humoral and cellular immune responses, findings that were corroborated by subsequent viral challenge experiments. To further evaluate protective efficacy, MPXV‐challenged BALB/c mice demonstrated full protection across all vaccine groups, with MV2 exhibiting superior performance in mitigating viremia and pathological damage. Given the orthopoxvirus homology between VACV and MPXV, we assessed cross‐protection using lethal‐dose VACV (30LD_50_) [13]. Through comprehensive monitoring of physiological parameters and multiorgan virological/pathological analyses, all four vaccines were shown to confer complete protection against lethal VACV challenge, indicating cross‐reactive immunity against orthopoxviruses. Pox lesion formation is a hallmark clinical feature of mpox infection. Recent studies have shown that interferon signaling‐deficient AGB6 mice develop prominent pox lesions following MPXV challenge. To further evaluate the efficacy of the vaccine against MPXV, we immunized AGB6 mice with the four candidate vaccines at a dose of 10 µg using the same regimen as previously described and assessed both its immunogenicity and protective efficacy. The results demonstrated that only the bivalent antigen vaccines MV1 and MV2, encoding both A35R and M1R, elicited protective effects, further confirming the critical importance of A35R and M1R in MPXV vaccine design. Although MV1 also reduced MPXV‐induced pathology in AGB6 mice, its protective efficacy was inferior to that of MV2, whether in reducing virus replication in blood and tissues or protecting organ tissues from damage.

Based on its superior immunogenicity and challenge protection, we sought to evaluate the long‐term immune response profile and protective efficacy of the MV2 vaccine to comprehensively assess its potential as a clinical vaccine candidate. We conducted a long‐term evaluation of mice immunized with the MV2 vaccine. Binding antibody levels were measured at multiple time points after primary immunization, while neutralizing antibody titers and cellular immune responses were assessed on Day 280 after primary immunization. Mice from the MV2‐10 µg group were subsequently subjected to a challenge protection experiment. The results demonstrated that the MV2 vaccine maintained durable humoral and cellular immunity for up to 280 days after primary immunization and conferred partial protection against MPXV in mice. Specifically, the vaccine partially reduced lung damage induced by intranasal MPXV challenge in BALB/c mice and significantly suppressed viral replication in lung tissues (Figure S16).

Although our designed vaccines demonstrated favorable efficacy, several limitations persisted. The investigation of vaccine antigen structures relied entirely on the AlphaFold3 protein structure prediction platform. While the predicted structures showed good confidence scores, their complete identity with authentic structures remains unverified, necessitating protein expression and structural validation through cryo‐electron microscopy (cryo‐EM). Although we conducted a comprehensive evaluation of the vaccines using the AGB6 mouse model capable of developing pox lesions, their physiological relevance remains inferior to that of nonhuman primate (NHP) models. These critical aspects will be addressed in future investigations.

We comprehensively evaluated the immunogenicity and protective efficacy of the four mRNA vaccines, and our systematic analysis demonstrated that the antigens A35R and M1R exhibited excellent immunogenicity, capable of eliciting robust humoral and cellular immune responses. In this study, the MV2 vaccine designed using a single‐chain “dimer‐like” strategy demonstrated superior immunogenicity and conferred enhanced protective efficacy against both MPXV and VACV in mice. These findings validated this design strategy as a feasible antigen development approach, providing novel insights for the clinical development of high‐efficacy vaccines against MPXV. Owing to its high immunogenicity and superior protective efficacy, the MV2 vaccine holds significant potential as a promising clinical candidate vaccine.

## 4 Materials and Methods

### 4.1 Animals and Ethics Statement

AGB6 and BALB/c mice aged 6–8 weeks were obtained from the Institute of Medical Biology, Chinese Academy of Medical Sciences. All the specimens were housed under specific pathogen‐free (SPF) barrier‐maintained conditions. Experimental protocols received rigorous review and approval from the Institutional Animal Care and Use Committee (IACUC) at the Institute of Medical Biology, Chinese Academy of Medical Sciences, with strict adherence to the NIH Guidelines for Laboratory Animal Welfare (NIH Publication No. 8023). VACV challenge studies were performed in certified Animal Biosafety Level 2 (ABSL‐2) containment laboratories at the aforementioned institute. Conversely, all MPXV infection protocols were implemented under enhanced biocontainment conditions meeting the Animal Biosafety Level 3 (ABSL‐3) standards at the same institution.

### 4.2 Cells and Viruses

The 293T and Vero cell lines were procured from established cryopreserved repositories within our institutional biospecimen bank. Both cellular systems were maintained in Dulbecco's modified Eagle's medium (DMEM, #8123187) supplemented with 10% heat‐inactivated fetal bovine serum (FBS, #2440086) and 1% penicillin‒streptomycin antibiotic cocktail under standard humidified incubation conditions (37°C, 5% CO_2_). MPXV (clade IIb) and vaccinia virus (VACV‐VTT) were obtained from the National Kunming High‐level Biosafety Primate Research Center, Institute of Medical Biology, Chinese Academy of Medical Sciences, and Peking Union Medical College. The experimentally adapted viral variant was generated via serial in vivo passaging in murine models during pathogenicity assessments. Both MPXV and VACV were propagated in Vero cell monolayers, with subsequent viral stocks processed into working aliquots and quantified as plaque‐forming units per milliliter using standard plaque assay methodology under methylcellulose overlay. Procedures involving viable pathogens were conducted at designated biocontainment facilities (VACV in BSL‐2 and MPXV (clade IIb) in BSL‐3) at the Institute of Medical Biology, Chinese Academy of Medical Sciences (IMBCAMS). MPXV (clade Ib) originated from the Guangdong Provincial Center for Disease Control and Prevention. Experiments involving MPXV (clade Ib) were conducted in the BSL‐3 facility at the Guangdong Provincial Center for Disease Control and Prevention.

### 4.3 LNP Morphology Characterization Using Cryo‐Electron Microscopy

With the assistance of Xi'an Jiaotong University, LNP morphology was characterized. Aliquots of LNPs (2.5 µL) were applied onto glow‐discharged Quantifoil Cu R1.2/1.3 (300 mesh) carbon‐supported films. Following a 5‐s blotting step using a Vitrobot Mark IV (Thermo Fisher Scientific), the samples were rapidly plunge‐frozen in liquid ethane. Image acquisition was performed using a Talos F200C transmission electron microscope equipped with a Ceta 4k×4k camera.

### 4.4 In Silico Prediction of Immunogen Structure

The “dimer‐like” structures of A35R and M1R were predicted using AlphaFold3 (https://alphafoldserver.com/) using the following protocol. First, the amino acid sequences of immunogens were input into the AlphaFold3 web interface, followed by structural modeling using default parameters. For antigen‒antibody docking predictions, the amino acid sequences of immunogens and antibody light/heavy chains were separately entered into designated input fields within AlphaFold3, with subsequent docking simulations performed under standard parameter settings. The highest scoring prediction was selected for structural visualization and analysis using PyMOL (v4.6.0), enabling detailed examination of molecular interactions and conformational characteristics.

### 4.5 Antibody Endpoint Titer Measurement With ELISA

Recombinant A35R protein and M1R protein (Antibodysystem, #EVV13101/#EVV13301, 1 µg/mL) were immobilized on 96‐well microplates (Thermo Fisher Scientific, #442404) via 16 h of adsorption at 4°C. Following three PBST (PBS with 0.05% Tween‐20) wash cycles under standardized conditions, nonspecific binding sites were blocked with 2% bovine serum albumin (BSA)/PBS through temperature‐controlled incubation (37°C, 60 min). Serum samples were subjected to serial dilution in assay buffer (0.05% BSA/PBST) at a 1:100 dilution ratio, followed by primary incubation (37°C, 60 min). Postwash procedures were repeated prior to the application of horseradish peroxidase‐conjugated polyclonal goat anti‐mouse IgG (Invitrogen, #A‐10668) at a 1:30,000 dilution in sample diluent (100 µL/well). Secondary incubation (37°C, 60 min) and subsequent washes preceded chromogenic development with 3,3',5,5'‐tetramethylbenzidine (TMB) substrate (Thermo Fisher Scientific, 100 µL/well) under ambient conditions (15 min). Enzymatic reactions were quenched using acidic stop solution (SolarBio, #C1058), with dual‐wavelength optical density measurements recorded at 450 nm (primary) and 630 nm (reference).

Negative control wells containing assay buffer in lieu of serum samples defined baseline values, while the seropositivity threshold was established at 2.1‐fold above the positive control wells. Geometric mean titers (GMTs) for antigen‐specific IgG were calculated as the maximal reciprocal dilution required to achieve threshold‐exceeding absorbance values.

### 4.6 Detection of Inflammatory Factors in Serum

Serum sampling was performed at 5‐ and 24‐h post‐immunization intervals. Quantitative cytokine profiling was conducted using the Bio‐Plex Pro Human Cytokine Screening Panel (Bio‐Rad Laboratories, #64521678) implemented on a Bio‐Plex 200 System (Bio‐Rad) in strict accordance with the manufacturer's standardized protocol.

### 4.7 ELISPOT Assay

The enzyme‐linked immunospot (ELISpot) assay was performed in accordance with the manufacturer's standardized protocol (Mabtech). Splenic tissues were aseptically dissected from murine subjects, followed by peripheral blood mononuclear cell (PBMC) isolation using a commercial lymphocyte separation kit (SolarBio, #P8860). Antigen‐specific stimulation was achieved through incubation with recombinant A35R or M1R protein (2 µg/50 µL per well), with experimental controls established as follows: baseline reactivity was assessed using unstimulated cells (negative control per experimental replicate), while phytohemagglutinin (PHA)‐activated cells served as positive process controls.

Subsequent steps included equilibration (37°C, 5% CO_2_), automated wash cycles, and cell seeding into precoated 96‐well ELISpot plates (Mabtech; #3321‐4APT‐10 membrane plates, #3441‐4APW‐10 plate covers). Final spot quantification and high‐resolution image acquisition were conducted using the IRIS Automated ELISpot Reader System (Mabtech), with the data normalized to spot‐forming units (SFU) per 10^6^ cells.

### 4.8 Isolation of IMV and EEV Particles From MPXV and Live MPXV and VACV Virus Neutralization Assays

MPXV stock was inoculated onto confluent monolayers of BSC‐1 cells in T75 flasks at an MOI of 0.5. After 2–3 days of incubation, when an approximately 90% cytopathic effect (CPE) was observed, the culture supernatant was discarded. Cells were scraped from the flasks, resuspended in 5–6 mL of PBS, and transferred to 15‐mL centrifuge tubes. The suspensions were subjected to three cycles of freeze‒thaw cycles on dry ice, followed by centrifugation at 5000 × *g* for 5 min at 4°C to remove cellular debris. The supernatant was then mixed thoroughly and aliquoted to obtain IMV particles. The MPXV stock was inoculated onto confluent monolayers of BSC‐1 cells in T75 flasks at an MOI of 0.5. After 2–3 days of incubation, when an approximately 90% CPE was observed, the cell culture supernatant was collected and centrifuged at 8000 × *g* for 10 min at 4°C to remove cellular debris. The clarified supernatant was then mixed thoroughly and aliquoted to obtain EEV particles. The serum was inactivated at 56°C for 30 min. Serial dilutions were prepared in deep‐well plates using DMEM, with eight dilution gradients ranging from 1:16 to 1:2048. The virus was diluted in DMEM to 1000 PFU/mL. Equal volumes of diluted virus solution were added to serially diluted serum samples, gently pipetted‐mixed, and incubated at 37°C with 5% CO_2_ for 1 h. The medium in the 24‐well plates was aspirated, followed by the addition of 200 µL of serum‐virus mixture per well (equivalent to 100 PFU/well). The plates were thoroughly shaken to ensure uniform viral distribution and incubated for 1 h. After incubation, the virus solution was aspirated, and 800–1000 µL of 0.9% methylcellulose semisolid overlay medium was added to each well. The plates were statically incubated at 37°C with 5% CO_2_ for 5–6 days. (Note: Overlay medium was added immediately after aspiration to prevent cell detachment. Three to four wells were aspirated at a time before immediate overlay addition.) After culture, the medium was aspirated, and the cells were fixed with 4% paraformaldehyde for 20–30 min. The fixative was removed, the plaques were stained with 1% crystal violet and counted, and the results were calculated.

Neutralization efficacy was determined through quantitative microscopic analysis of virus‐induced CPE progression. Serum neutralization titers were computed as the reciprocal endpoint dilution achieving ≥50% CPE inhibition relative to the virus control wells and are expressed as 50% neutralization titers (NT_50_).

### 4.9 MPXV and VACV Challenge

Using an intranasal challenge approach, mice were administered either 1 × 10^6^ PFU MPXV or 30 LD50 of VACV. In MPXV‐challenged mice, body temperature and body weight were monitored every other day, and blood collection and oropharyngeal swab sampling were performed. All the animals survived until Day 7 post‐challenge and were subsequently euthanized. Cardiac, hepatic, splenic, pulmonary, renal, cerebral, duodenal, and rectal tissues were harvested for subsequent viral load quantification and histopathological analysis. In VACV‐challenged mice, body temperature and body weight measurements were recorded every other day. All control group mice died by post‐challenge Day 6, with terminal body weight and temperature documented. Heart, liver, spleen, lung, kidney, brain, duodenum, and rectum specimens were collected from deceased controls. All vaccinated cohorts survived through post‐challenge Day 12 prior to euthanasia. The identified visceral organs (hearts, livers, spleens, lungs, kidneys, brains, duodenums, and rectums) were harvested from the mice in the vaccine group for subsequent viral load quantification and histopathological analysis.

### 4.10 Quantification of Viral Load in Mice

Viral RNA burden quantification in the blood and organs of virus‐challenged mice was performed via quantitative reverse transcription–PCR (RT‒qPCR). Whole blood samples were homogenized with TRIzol LS reagent (1:3 v/v ratio), whereas tissue samples (100–200 mg wet mass) were mechanically homogenized in 800 µL of TRIzol reagent (Thermo Fisher Scientific) to ensure complete viral inactivation. RNA was extracted from 200 µL of TRIzol lysates using the U‐Pure Virus RNA Plus Kit (BioKeystone Technologies, #M2006P‐A96), and the resulting nucleic acid eluates were subjected to downstream molecular analysis. Amplification reactions were conducted on a CFX384 Touch Real‐Time PCR System (Bio‐Rad Laboratories) employing TaqMan Fast Virus 1‐Step Master Mix (Thermo Fisher Scientific, #4444432). The following target‐specific oligonucleotide primers and dual‐quenched hydrolysis probes were designed to target the viral structural protein‐encoding gene:

MPXV‐F: GGAAAATGTAAAGACAACGAATACAG
MPXV‐R:GCTATCACATAATCTGGAAGCGTA
VACV‐F: CGGCTAAGAGTTGCACATCCA
VACV‐R:CTCTGCTCCATTTAGTACCGATTCT
MPXV‐Probe: FAM‐AAGCCGTAATCTATGTTGTCTATCGTGTCC‐BHQ1
VACV‐prob: FAM‐AGGACGTAGAATGATCTTGTA‐BHQ1

Quantitative standardization was achieved through serial 10‐fold dilutions of recombinant plasmid DNA carrying the target amplicon. The one‐step thermocycling parameters included the following: reverse transcription: 25°C × 2 min → 50°C × 15 min; initial denaturation: 95°C × 2 min; amplification (40 cycles): denaturation: 95°C × 5 s; and annealing/extension: 60°C × 31 s.

### 4.11 Histopathology

The tissue samples were immersed in 4% paraformaldehyde (PFA) for 72 h, followed by standard paraffin‐embedding processing. Serial 5‐µm‐thick sections were generated using a microtome for subsequent hematoxylin and eosin (H&E) histochemical staining. Whole‐slide imaging was performed utilizing a 3DHISTECH high‐resolution digital scanning system. Histopathological evaluation was conducted by certified veterinary pathologists through computerized morphometric analysis implemented in the manufacturer‐specific CaseViewer platform, employing standardized semiquantitative scoring criteria for inflammatory infiltrates and tissue architecture alterations. Specifically, for lung tissues, the evaluation focused on the presence or absence of thickening of the interalveolar septa, hemorrhage in the septa and alveoli, thrombosis, exudates and obstruction in the bronchi, inflammatory cell infiltration, dust‐laden macrophage distribution, and carbon particle deposition. For liver tissues, hepatocyte swelling, congestion or hemorrhage, and lymphocyte infiltration were assessed. For brain tissues, examination focused on hemorrhage, microgliosis, neuronal necrosis, and the presence of dust‐laden macrophages with carbon particle deposition. With respect to the spleen tissue, the analysis revealed scattered transparent vascular follicles, active germinal centers, loss of germinal centers, lymphocyte infiltration, and diffuse hemorrhage. For kidney tissues, the evaluation focused on hemorrhage, inflammatory cell infiltration, and exudates. For duodenal tissues, assessments included localized hemorrhage, mucosal sloughing, and inflammatory cell infiltration. For cardiac tissues, the examination revealed localized congestion or hemorrhage, inflammatory cell infiltration, and myocardial fibrosis. For rectal tissues, the evaluation focused on localized hemorrhage, mucosal sloughing, and inflammatory cell infiltration. The scale bar represents 200 µm.

### 4.12 Statistical Analysis

All the data are presented as the mean ± SEM. Statistical analyses were performed using GraphPad Prism software (CA, USA). For multiple‐group comparisons (three or more groups), one‐way ANOVA and Tukey's multiple comparison test were used for bar graphs; *p < 0.05; **p < 0.01; ***p < 0.001; ****p < 0.0001; ns, not significant.

## Author Contributions

Cong Tang, Yun Yang, Junbing Wang, and Wenhai Yu vaccinated mice for immunization and performed the experimental study and evaluation of mouse antiviral responses. Shuaiyao Lu and Youchun Wang provided research design and financial support. Shuaiyao Lu, Longhai Yuan, and Cong Tang performed selection of vaccine antigen and sequence design. Yun Yang, Yanan Zhou, and Qing Huang were associated with determination of virus load and neutralizing antibody. Longhai Yuan, Rui Peng, and Jiali Xu performed evaluation of immunogenicity of the vaccines and data collection. Yun Xie performed observation of LNP forms. Baisheng Li provided partial strains for the experiments. Shuaiyao Lu and Wenqi Quan managed funding. Longhai Yuan, Shuaiyao Lu, and Cong Tang were associated with figure generation and manuscript writing. All authors have read and approved the final manuscript.

## Funding

This work was supported partially by grants from the Science and Technology Leading Talent Program of Yunnan Province (202405AB350002), the High‐Level Medical Talent Training Program of Yunnan Province (L‐2024018), the Guangdong Provincial Center for Disease Control and Prevention Supports Talent Projects (0720240122), the CAMS Innovation Fund for Medical Sciences (2021‐I2M‐1‐038 and 2023‐I2M‐2‐001), and the Key Project of Basic Research in Yunnan Province (202401AS070049).

## Ethics Statement

All animal experiments were approved by the Institutional Animal Care and Use Committee of the Institute of Medical Biology, Chinese Academy of Medical Science (Ethics number DWSP202503063).

## Conflicts of Interest

The authors declare no conflicts of interest.

## Supporting information




**Figure S1**: MPXV clade II A35R‐A27D7/M1R‐7D11 antigen‐antibody docking prediction.
**Figure S2**: Antigen‐antibody docking prediction.
**Figure S3**: Antigen‐antibody docking prediction site.
**Figure S4**: “Dimer‐like” antigens.
**Figure S5**: dsRNA detection and LNP stability testing.
**Figure S6**: The results of in vitro expression of vaccine, complete Western Blot results.
**Figure S7**: Spot images from the Elispot IFN‐γ assay using different proteins as stimuli.
**Figure S8**: Spot images from the Elispot IL‐2 assay using different proteins as stimuli.
**Figure S9**: Changes in body temperature and detection of viral load in throat swabs in the MPXV challenge experiment in Balb/C mice.
**Figure S10**: Pathological damage and scoring in the heart, liver and brain of mice post‐MPXV challenge.
**Figure S11**: Pathological damage and scoring in the duodenum and rectum of Balb/c mice post‐MPXV challenge.
**Figure S12**: Pathological damage and scoring in the heart, brain and spleen of Balb/c mice post‐VACV challenge.
**Figure S13**: Pathological damage and scoring in the duodenum and rectum of Balb/c mice post ‐ VACV challenge.
**Figure S14**: Immunogenicity assessment of the vaccines in AGB6 mice.
**Figure S15**: Pathological damage and poxvirus shedding in AGB6 mice infected with MPXV.
**Figure S16**: Long‐term immunogenicity and vaccine efficacy of MV2 in 280 days post‐vaccination protection.

## Data Availability

The data generated or analyzed during the current study are available from the corresponding author upon reasonable request.
