# Supplementary material for: An mRNA Vaccine Based on Antigens From Conserved Regions of Monkeypox Virus A35R and M1R With a Dimer‐Like Conformation Confers Protection Against Both Monkeypox Virus and Vaccinia Virus Infections in Mice
Source: MedComm (2020). 2026 Jan 22;7(2):e70614. doi: 10.1002/mco2.70614 (PMC12828169; doi:10.1002/mco2.70614)
Supplement: Supplementary file 1 — Figure S1: MPXV clade II A35R‐A27D7/M1R‐7D11 antigen‐antibody docking prediction. Figure S2: Antigen‐antibody docking prediction. Figure S3: Antigen‐antibody docking prediction site. Figure S4: “Dimer‐like” antigens. Figure S5: dsRNA detection and LNP stability testing. Figure S6: The results of in vitro expression of vaccine, complete Western Blot results. Figure S7: Spot images from the Elispot IFN‐γ assay using different proteins as stimuli. Figure S8: Spot images from the Elispot IL‐2 assay using different proteins as stimuli. Figure S9: Changes in body temperature and detection of viral load in throat swabs in the MPXV challenge experiment in Balb/C mice. Figure S10: Pathological damage and scoring in the heart, liver and brain of mice post‐MPXV challenge. Figure S11: Pathological damage and scoring in the duodenum and rectum of Balb/c mice post‐MPXV challenge. Figure S12: Pathological damage and scoring in the heart, brain and spleen of Balb/c mice post‐VACV challenge. Figure S13: Pathological damage and scoring in the duodenum and rectum of Balb/c mice post ‐ VACV challenge. Figure S14: Immunogenicity assessment of the vaccines in AGB6 mice. Figure S15: Pathological damage and poxvirus shedding in AGB6 mice infected with MPXV. Figure S16: Long‐term immunogenicity and vaccine efficacy of MV2 in 280 days post‐vaccination protection. [file MCO2-7-e70614-s001.docx]

**Supplemental Information**

**An mRNA vaccine based on antigens from conserved regions of Monkeypox virus A35R and M1R with a dimer-like conformation confers protection against both monkeypox virus and vaccinia virus infections in mice**

Cong Tang^1#^, Longhai Yuan^1#^, Yun Xie^1#^,Yun Yang^1#^,Yanan Zhou^1^,Junbing Wang^1^, Hao Yang^1^,Rui Peng^1^, Jiali Xu^1^,Wenhai Yu^1^,Qing Huang^1^,Wenqi Quan^1^, Baisheng Li^2*^, Youchun Wang^1,3,4*^，Shuaiyao Lu^1,3,4,5,6*^

**This file includes:**

**Figure S1-Figure S16**

**Figure S1**

**
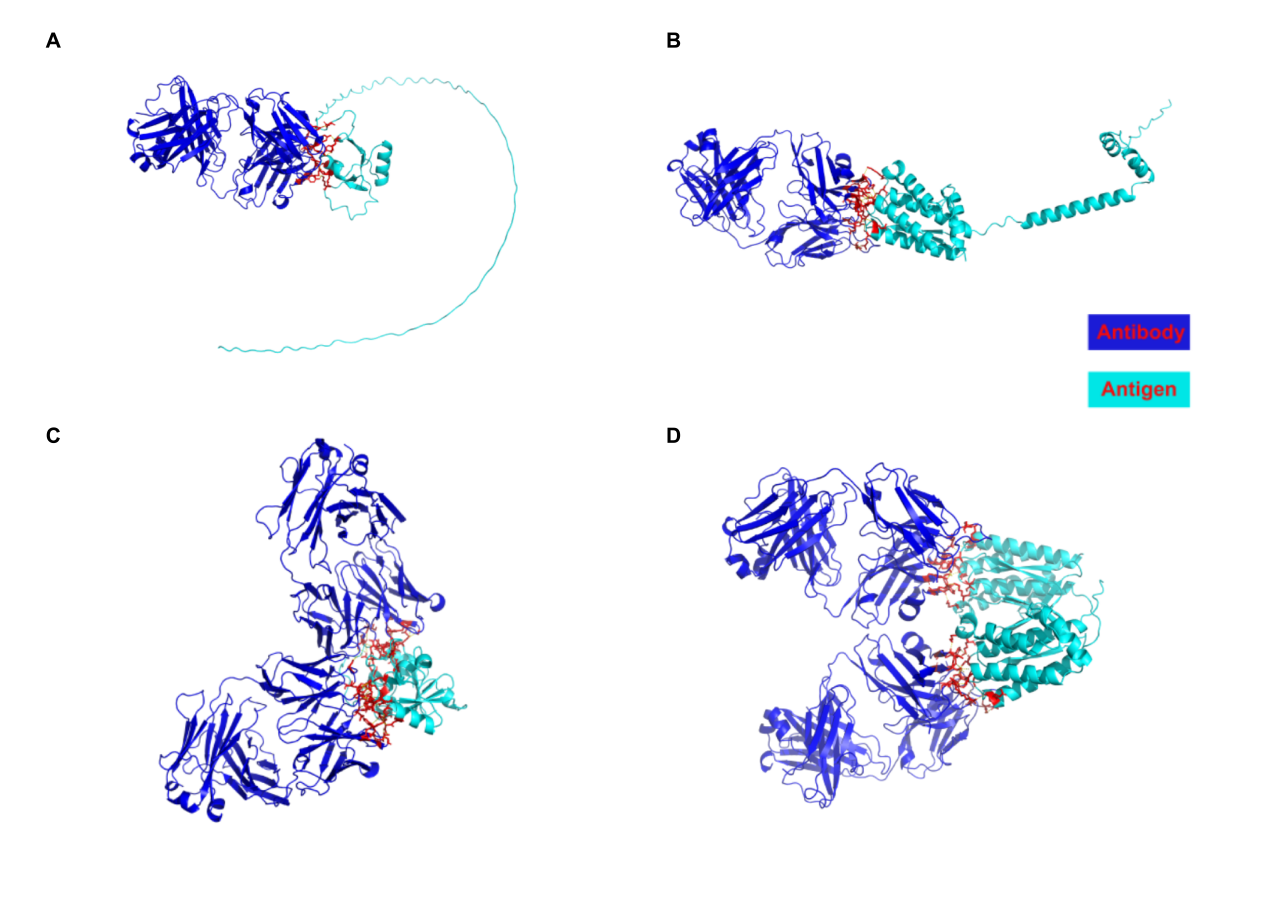
**

**Figure S1 MPXV clade II A35R-A27D7/M1R-7D11 antigen-antibody docking prediction**

1. Antigen-antibody docking prediction between A35R and A27D7.

B. Antigen-antibody docking prediction between M1R and 7D11.

C. Antigen-antibody docking prediction between “Dimer-like” A35R conformation and A27D7.

D. Antigen-antibody docking prediction between “Dimer-like” M1R conformation and 7D11.

**Figure S2**


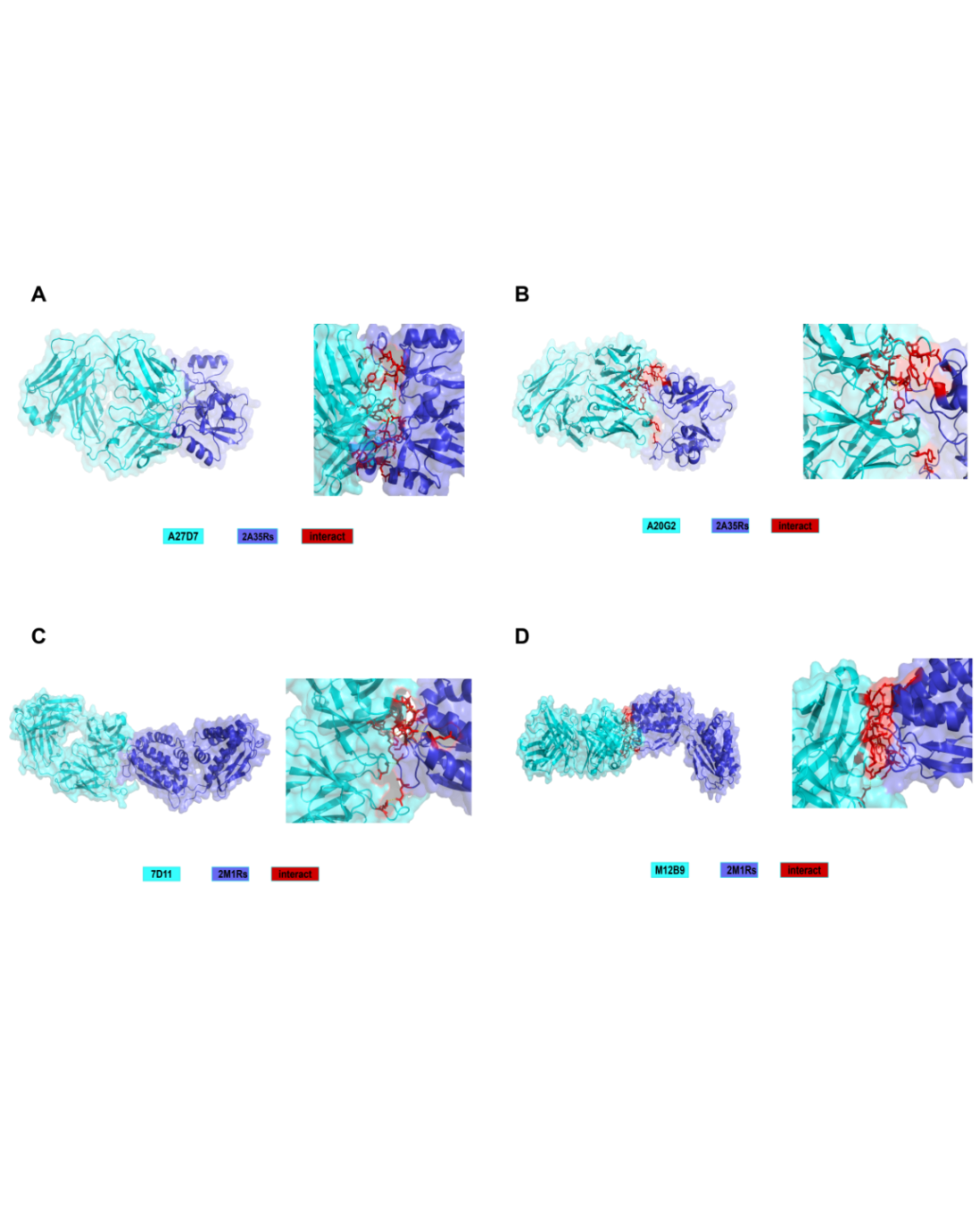


**Figure S2 Antigen-antibody docking prediction**

A,B. Antigen-antibody docking prediction between the "Dimer-like" conformation of A35R and protective antibodies A27D7/A20G2.

C,D. Antigen-antibody docking prediction between the "Dimer-like" conformation of M1R and protective antibodies 7D11/M12B9.

**Figure S3**


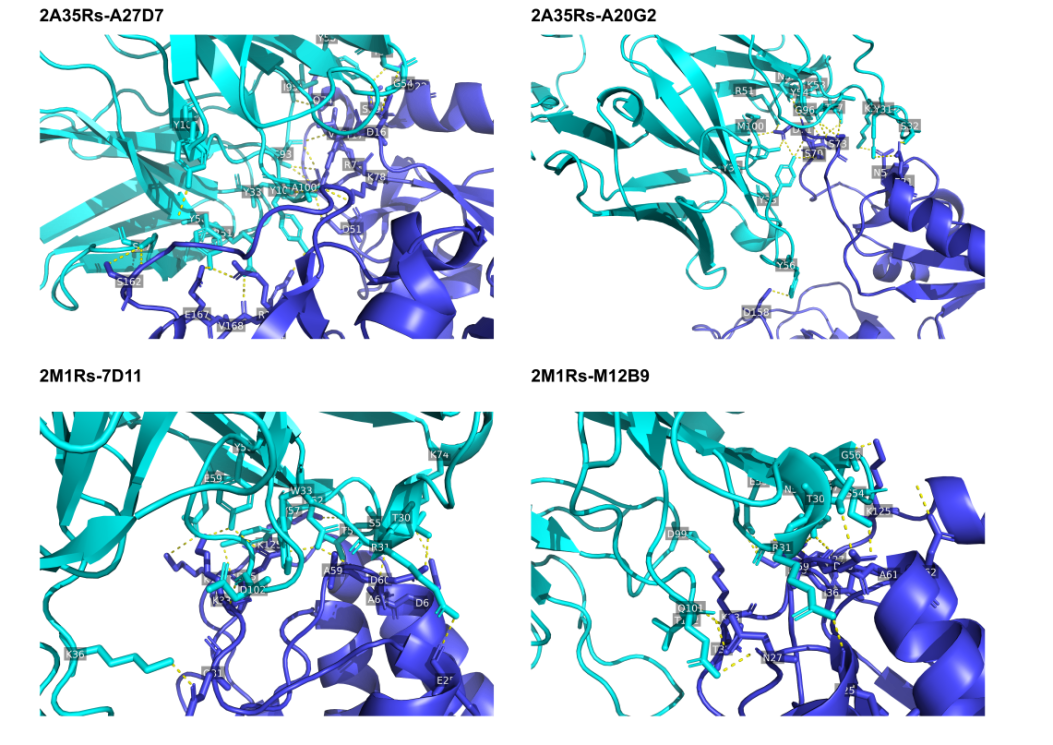


**Figure S3** **Antigen-antibody docking prediction site**

**Figure S4**
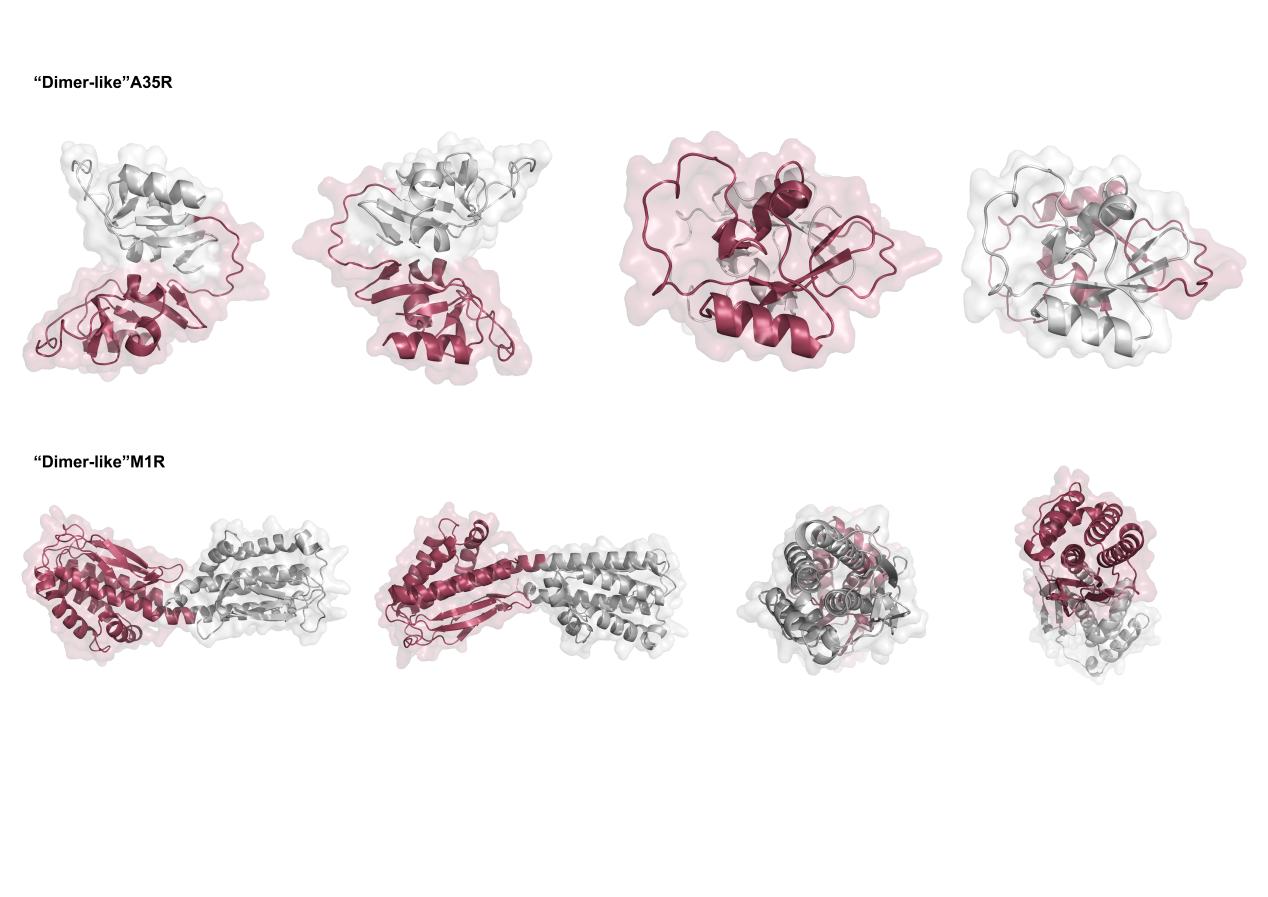


**Figure S4 “Dimer-like” antignes**

**Figure S5**


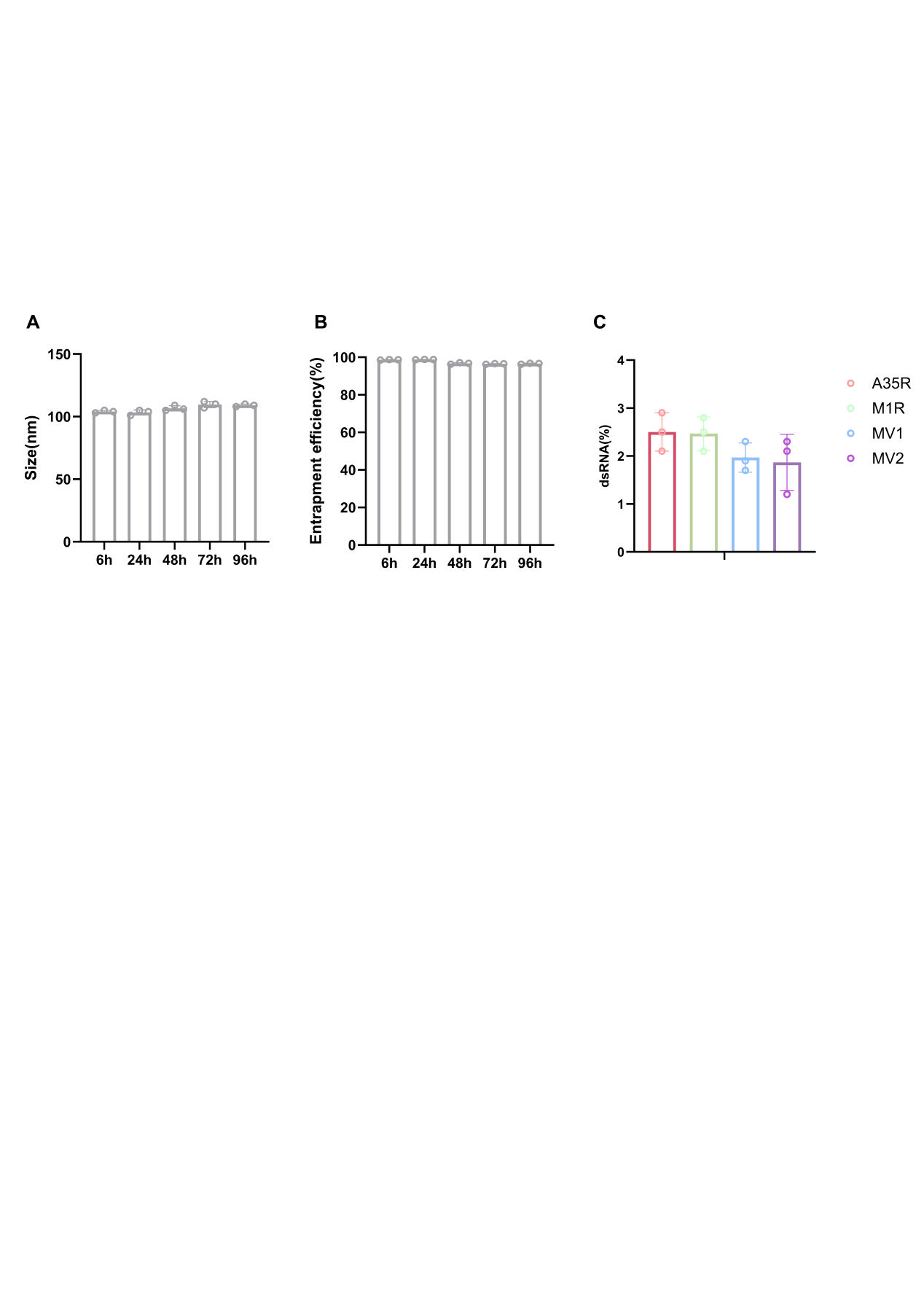


**Figure S5 dsRNA detection and LNP stability testing**

1. Particle size within 6 to 96 hours after vaccine preparation (repeated three times).
2. Encapsulation efficiency within 6 to 96 hours after vaccine preparation (repeated three times).
3. dsRNA content of four mRNA (three replicate measurements).

**Figure S6**

**
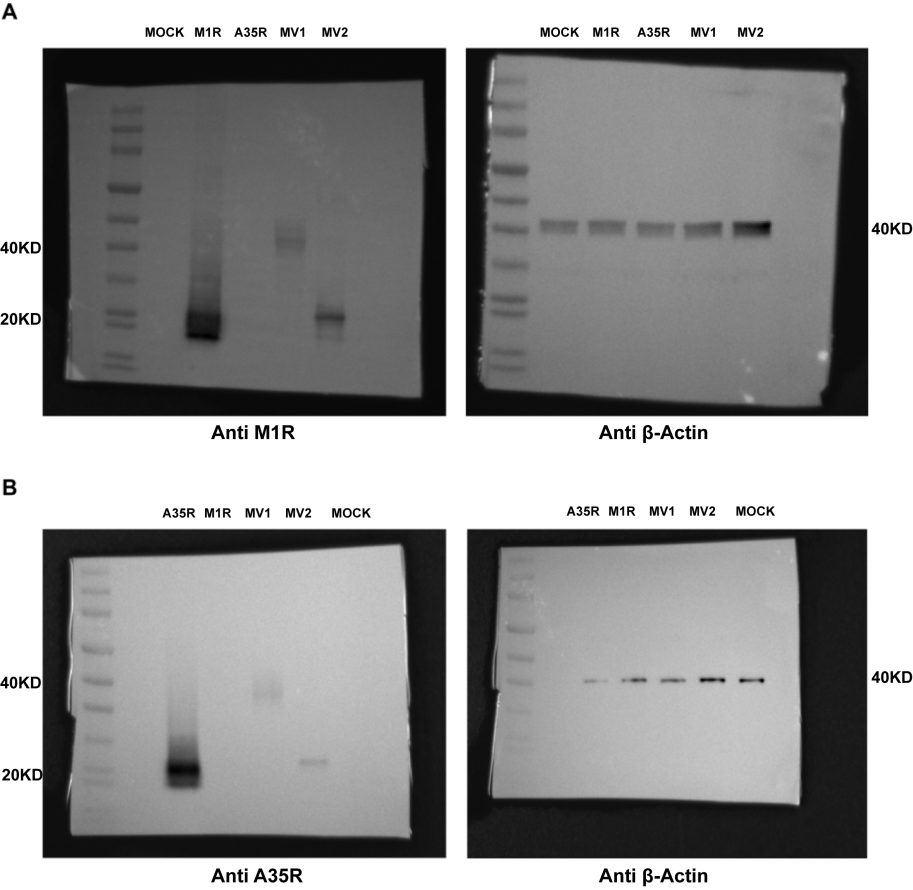
**

**Figure S6 The results of in vitro expression of vaccine, complete Western Blot results**

1. Following transfection of 293T cells with mRNA-LNP, proteins were harvested and subjected to Western blot analysis using an anti-M1R antibody.
2. Following transfection of 293T cells with mRNA-LNP, proteins were harvested and subjected to Western blot analysis using an anti-A35R antibody.

**Figure S7**

**
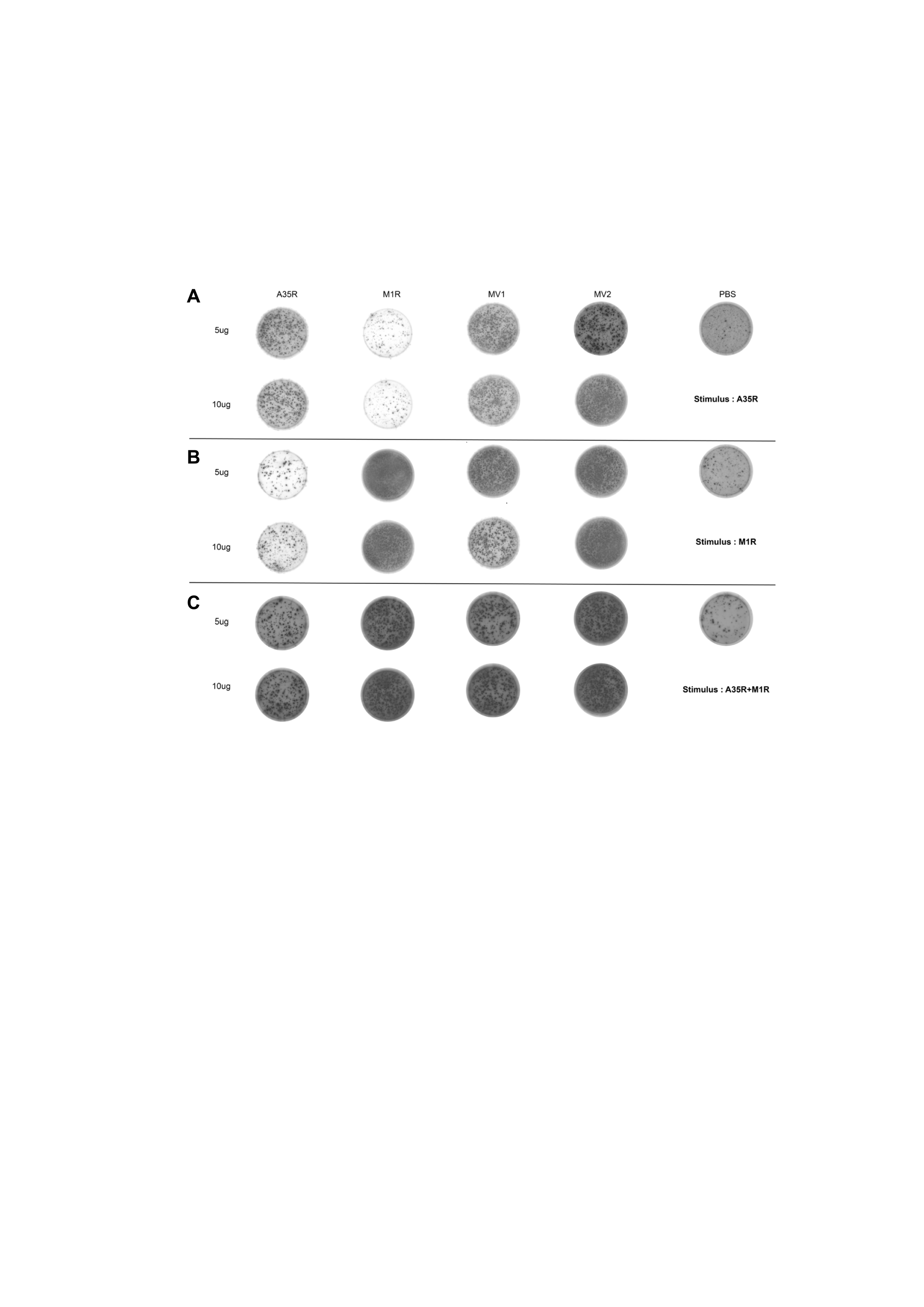
**

**Figure S7 Spot images from the Elispot IFN-γ assay using different proteins as stimuli**

1. Spot images from the ELISpot IFN-γ assay using A35R protein as the stimulus.
2. Spot images from the ELISpot IFN-γ assay using M1R protein as the stimulus.
3. Spot images from the ELISpot IFN-γ assay using A35R and M1R proteins as the stimulus.

**Figure S8
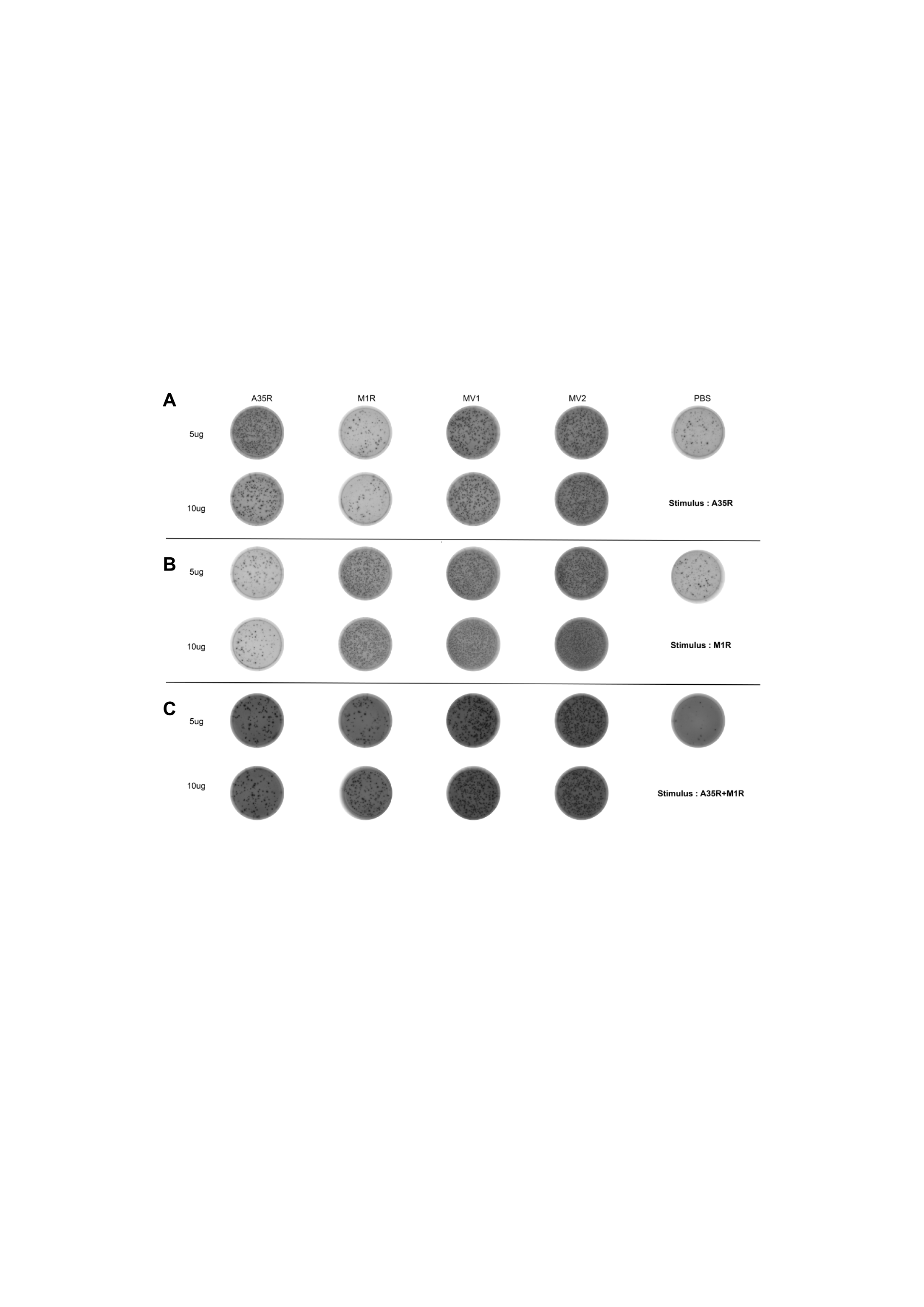
**

**Figure S8 Spot images from the Elispot IL-2 assay using different proteins as stimuli**

1. Spot images from the ELISpot IL-2 assay using A35R protein as the stimulus
2. Spot images from the ELISpot IL-2 assay using M1R protein as the stimulus
3. Spot images from the ELISpot IL-2 assay using A35R and M1R proteins as the stimulus

**Figure S9**

**
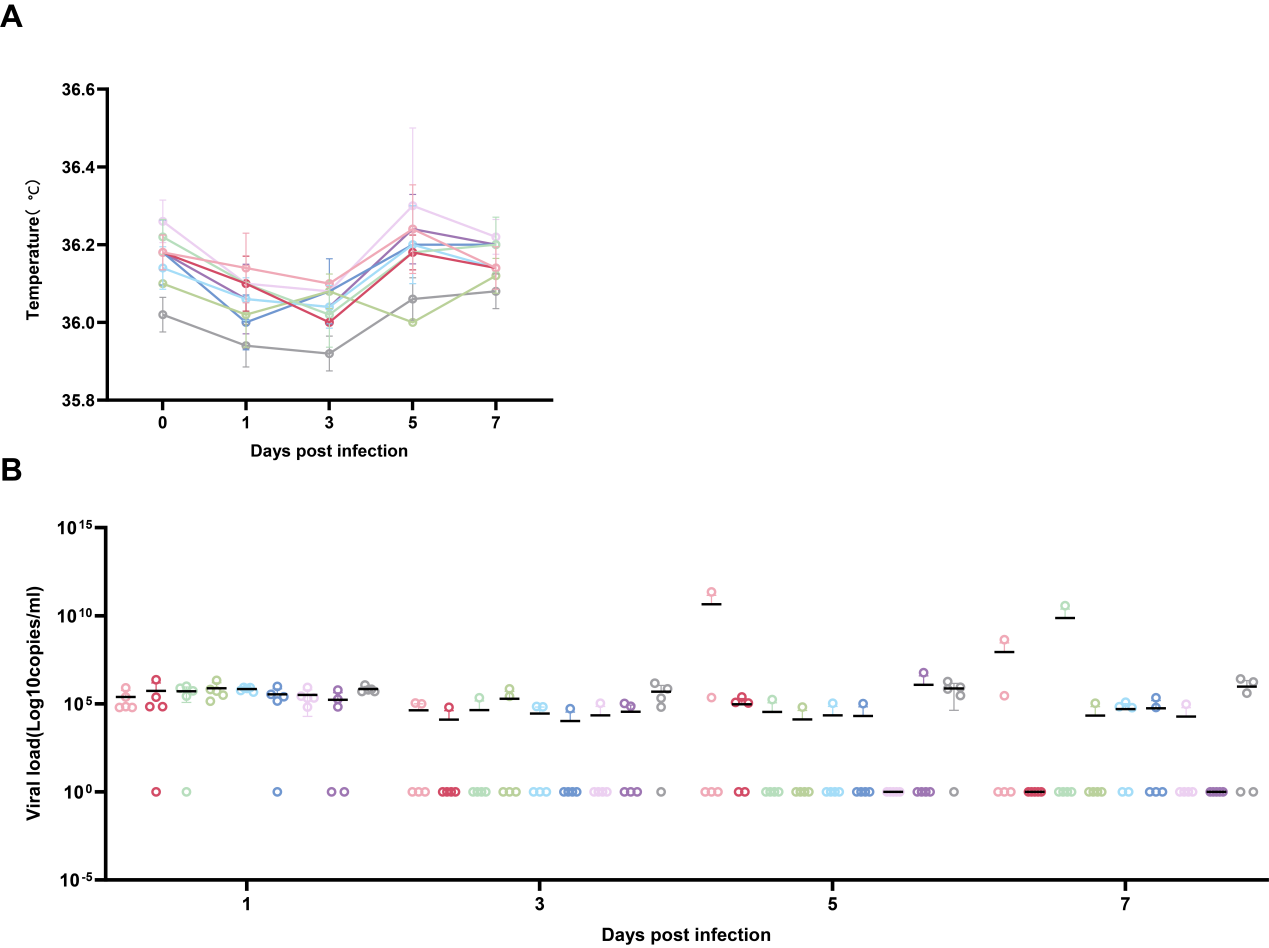
**

**Figure S9 Changes in body temperature and detection of viral load in throat swabs in the MPXV challenge experiment in Balb/C mice**

1. Temperature Change
2. Viral load of throat swabs

**Figure S10**


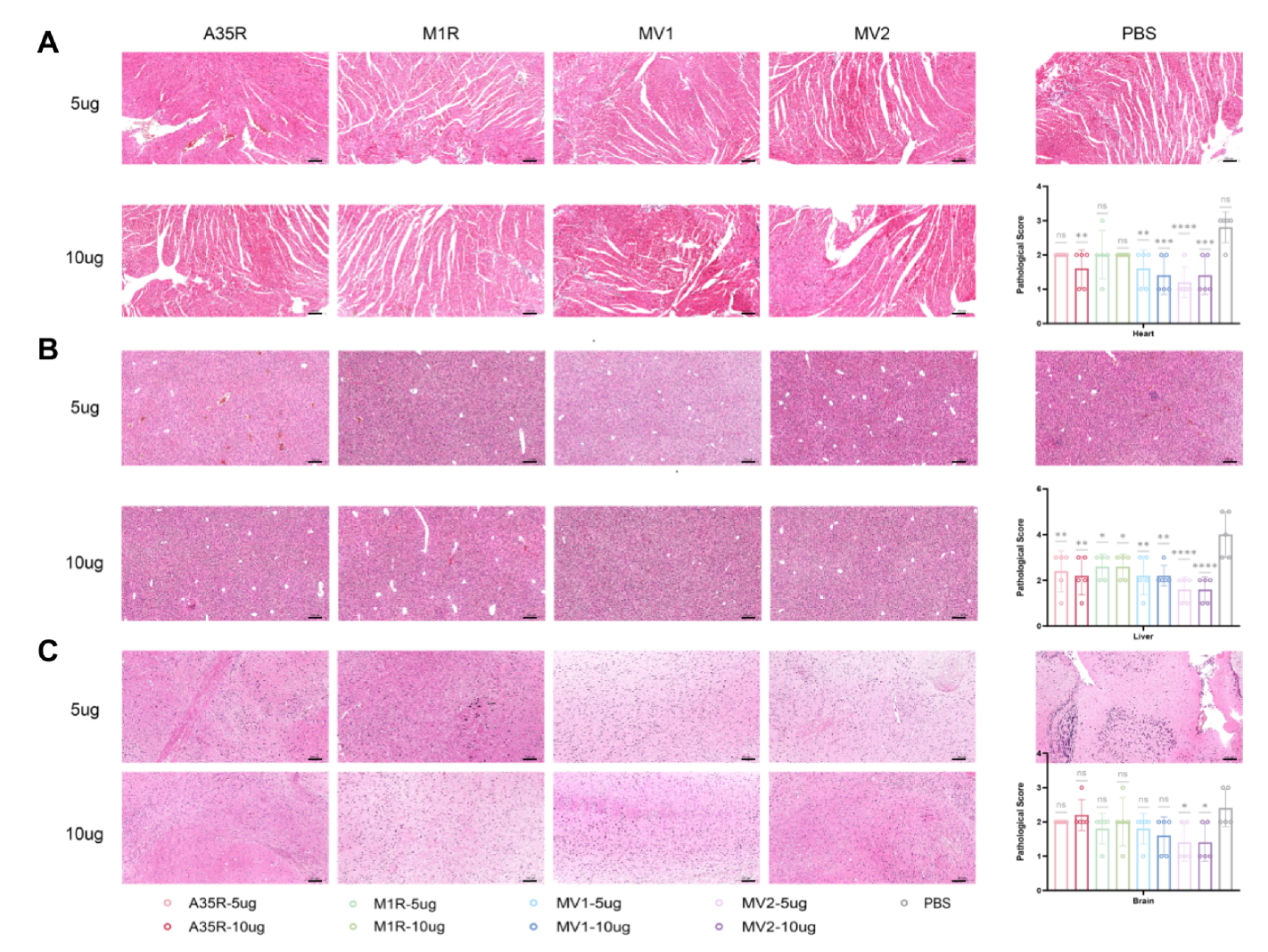


**Figure S10 Pathological damage and scoring in the heart, liver and brain of mice post-MPXV challenge**

1. Pathological damage and scoring in the heart.
2. Pathological damage and scoring in the liver.
3. Pathological damage and scoring in the brain.

**Figure S11**


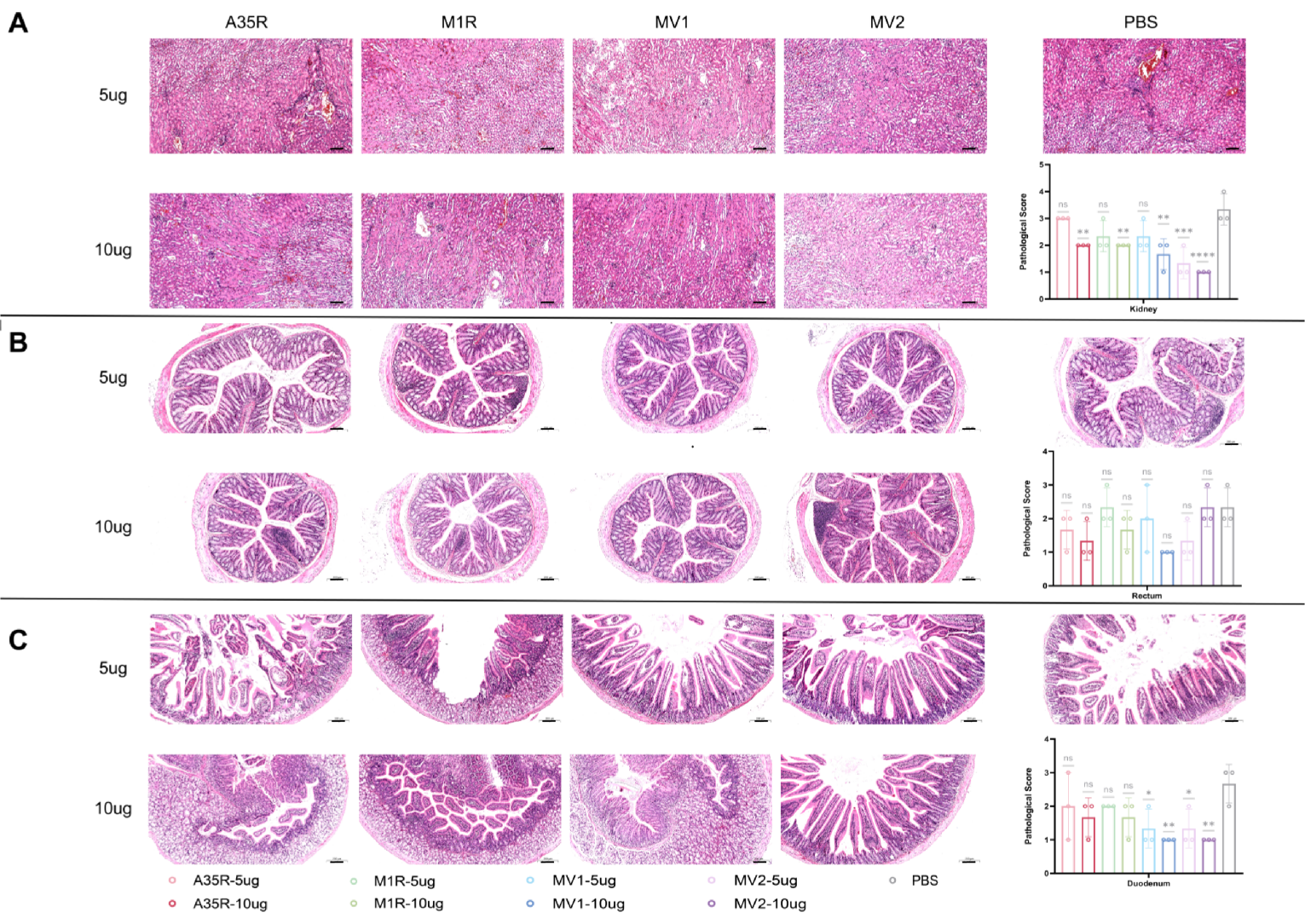


**Figure S11 Pathological damage and scoring in the duodenum and rectum of Balb/c mice post-MPXV challenge**

1. Pathological damage and scoring in the kidney.
2. Pathological damage and scoring in the rectum.
3. Pathological damage and scoring in the duodenum.

**Figure S12
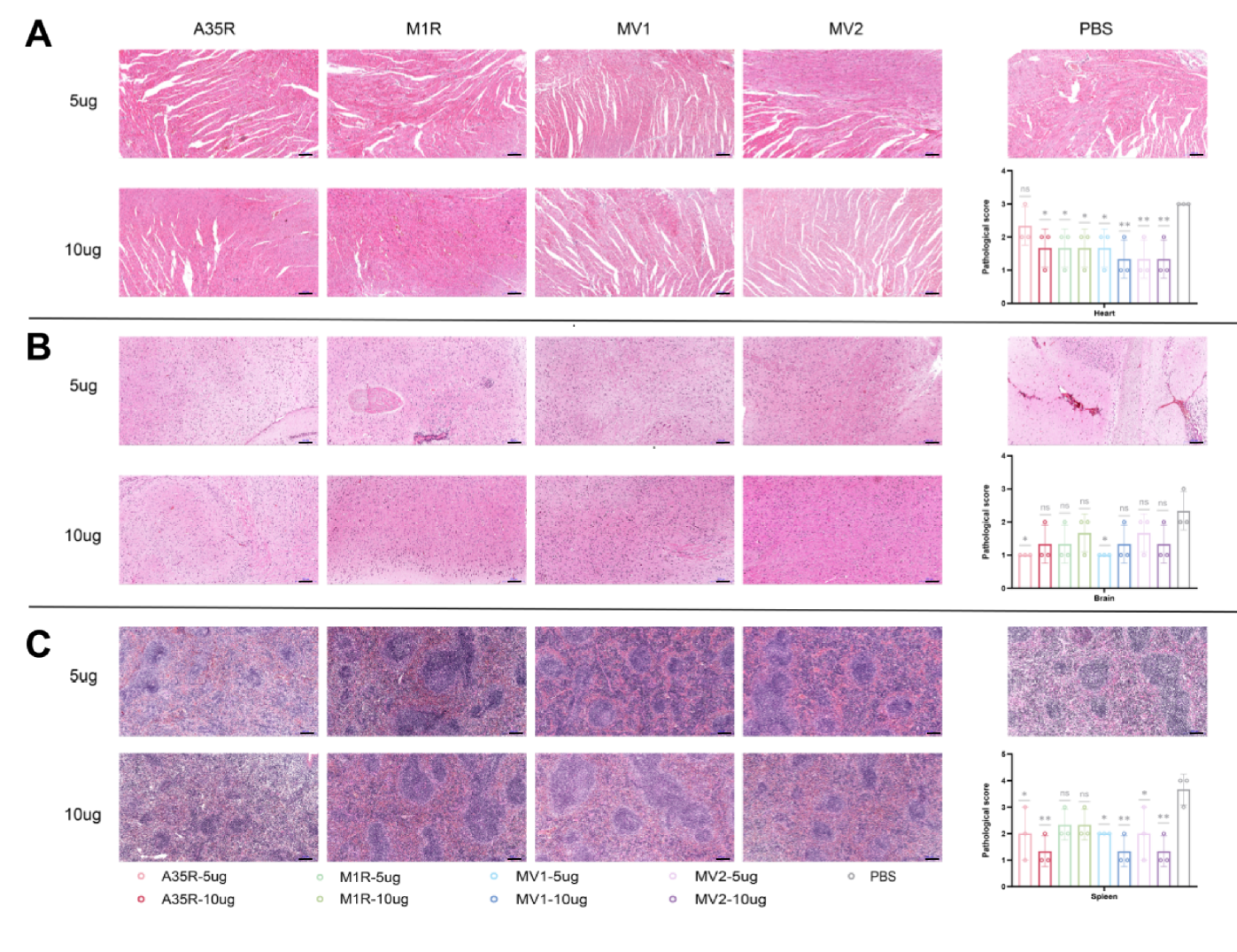
**

**Figure S12 Pathological damage and scoring in the heart,brain and spleen of Balb/c mice post-VACV challenge**

1. Pathological damage and scoring in the heart.
2. Pathological damage and scoring in the brain.
3. Pathological damage and scoring in the spleen.

**Figure S13**

**
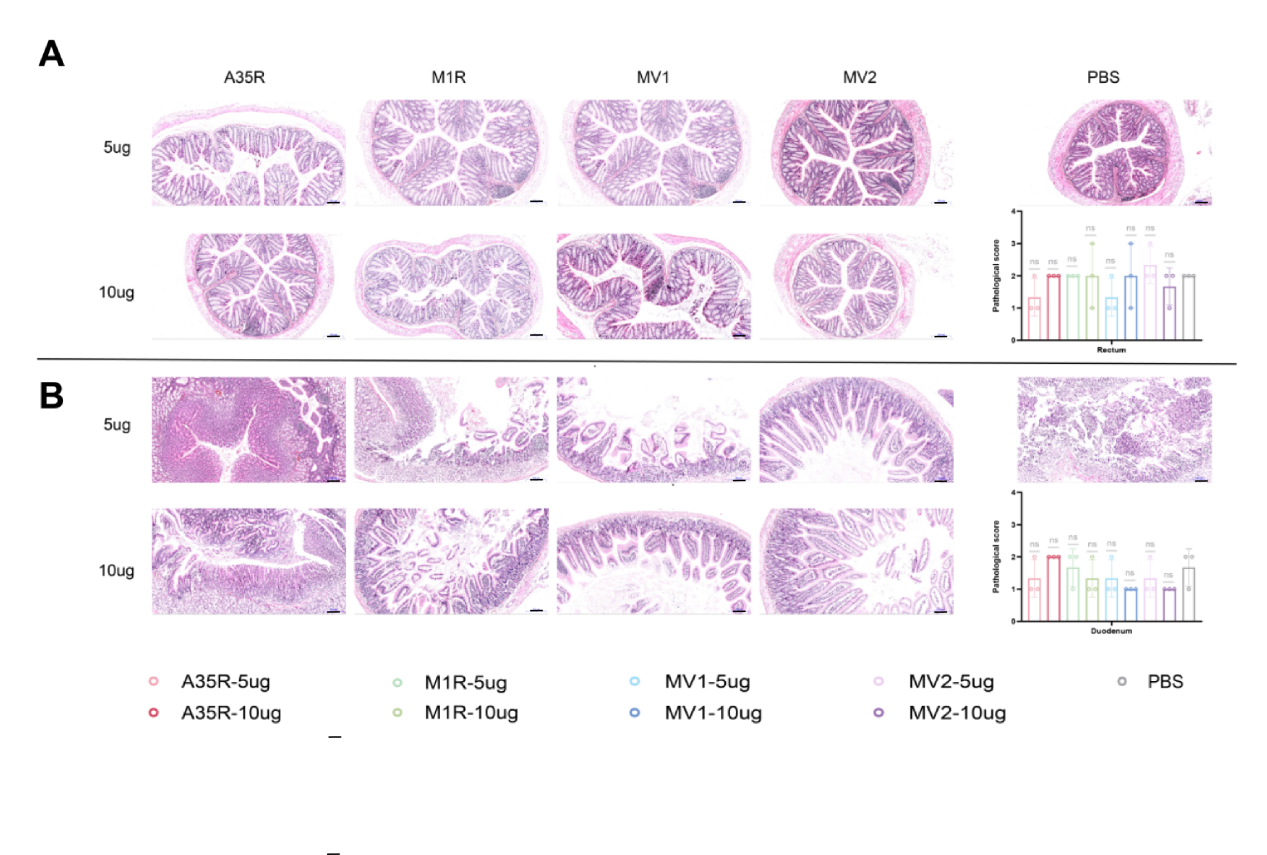
**

**Figure S13 Pathological damage and scoring in the duodenum and rectum of Balb/c mice post-VACV challenge**

1. Pathological damage and scoring in the rectum.
2. Pathological damage and scoring in the duodenum.

**Figure S14**


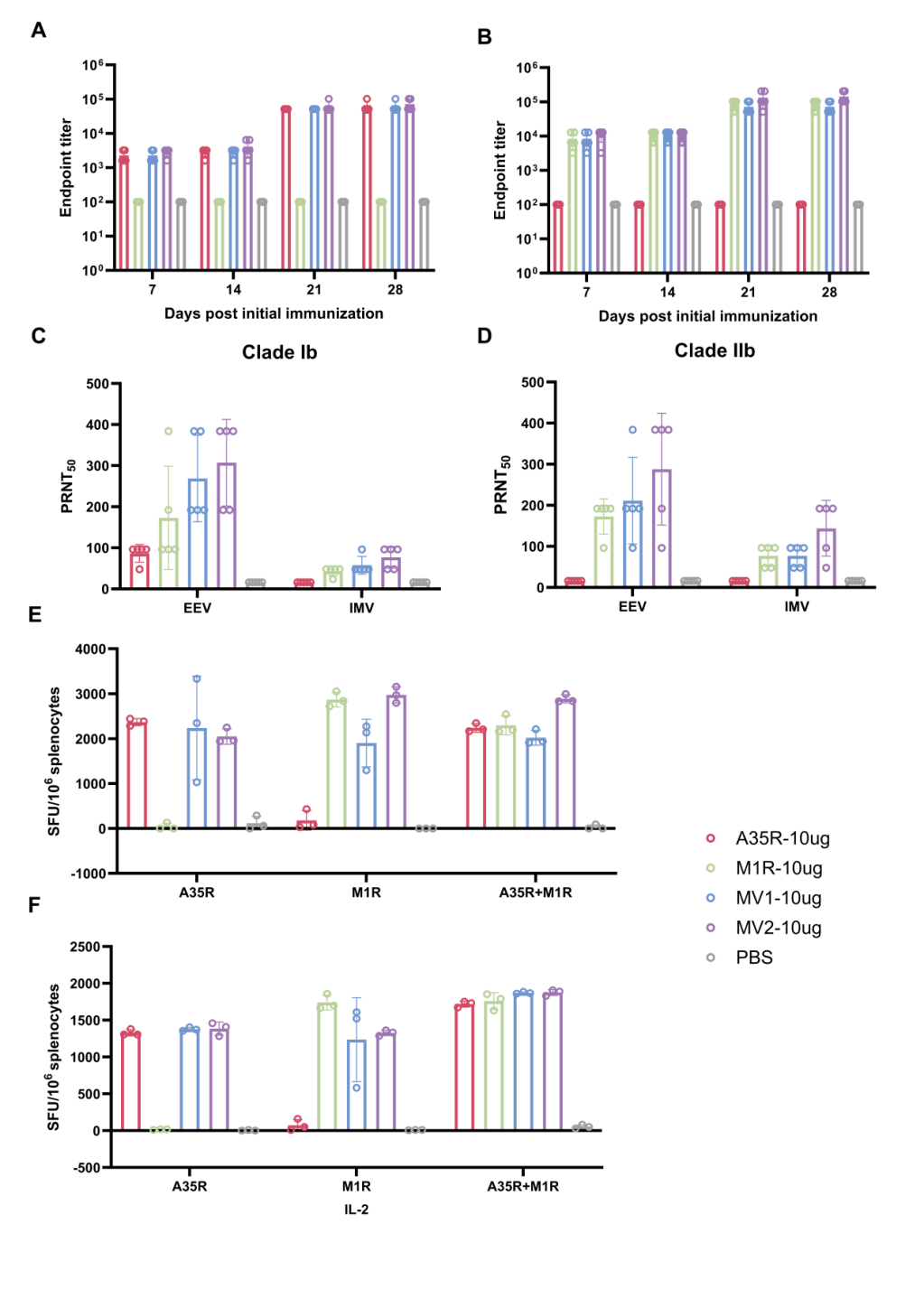


**Figure S14 Immunogenicity assessment of the vaccine in AGB6 mice**

1. Specificity of the Binding antibody titers against A35R.
2. Specificity of the Binding antibody titers against M1R.
3. Neutralizing antibody levels against MPXV Clade Ib.
4. Neutralizing antibody levels against MPXV Clade IIb.
5. Detection of IFN-γ Secretion by Elispot Assay.
6. Detection of IL-2 Secretion by Elispot Assay.

**Figure S15**


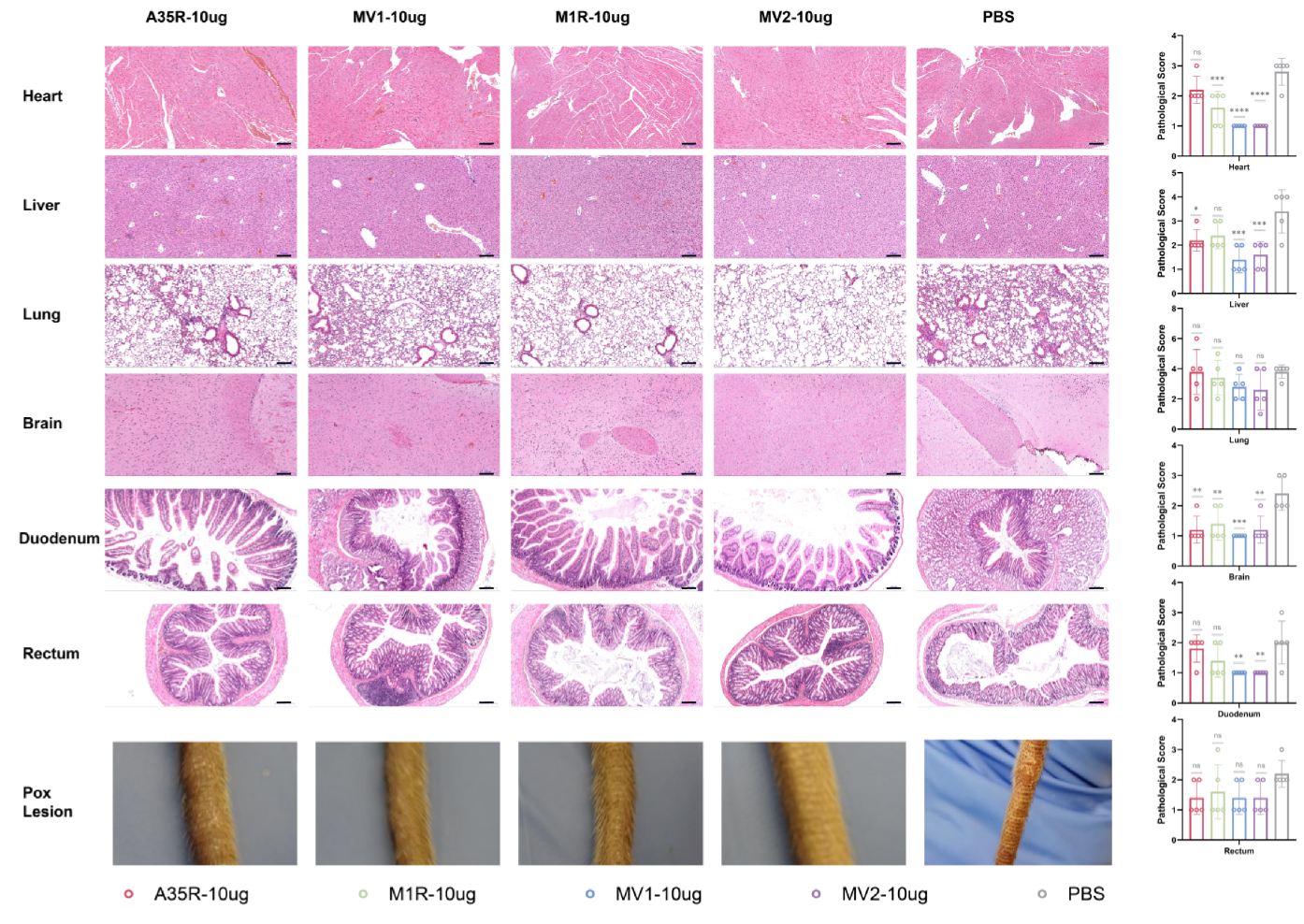


**Figure S15 Pathological damage and poxvirus shedding in AGB6 mice infected with MPXV**

**Figure S16**
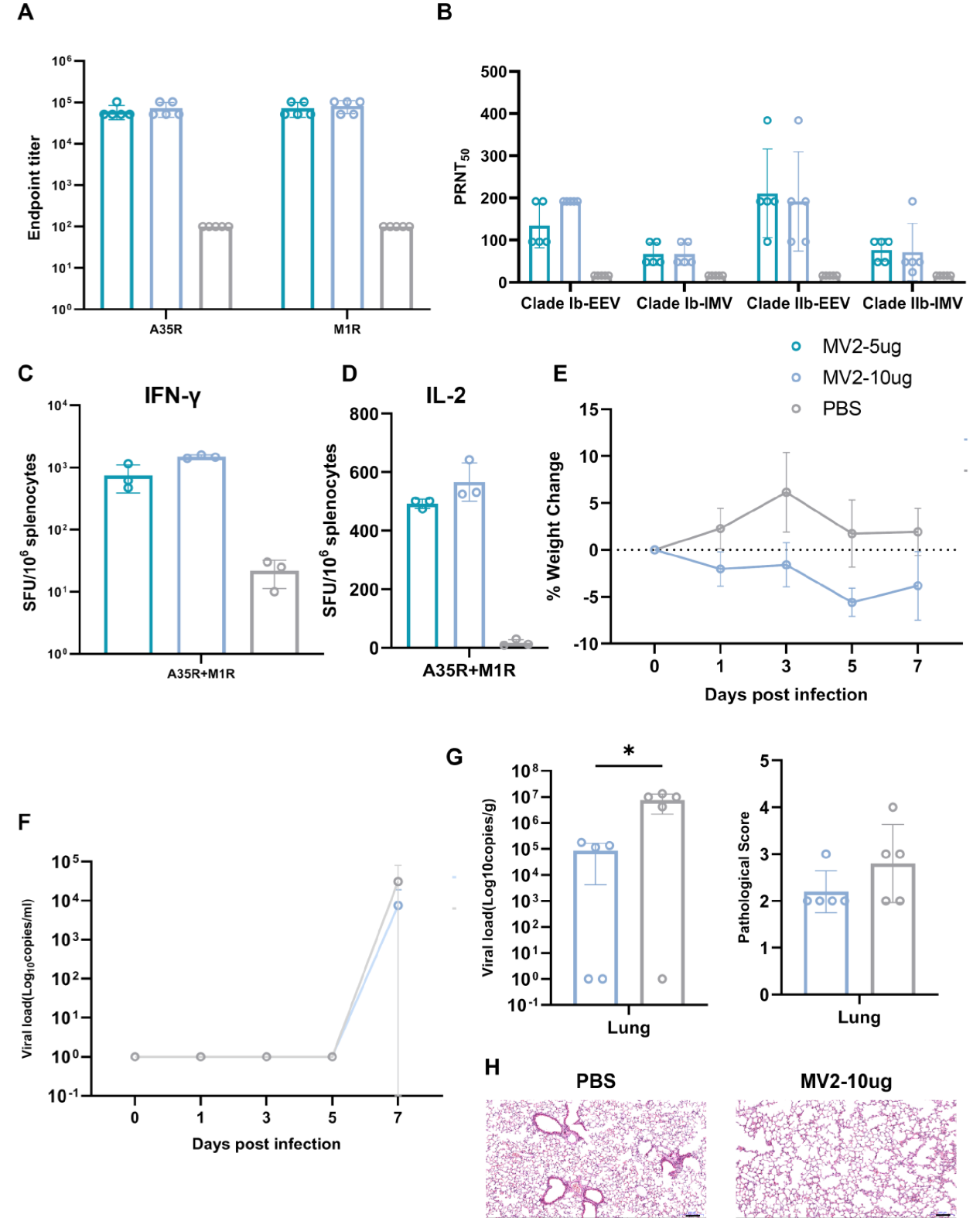


**Figure S16 Long-term immunogenicity and vaccine efficacy of MV2 in 280 day post-vaccination protection**

1. Binding antibody titers.
2. Neutralizing antibody level.
3. D. The number of IFN-γ and IL-2 spots produced by the spleen of mice stimulated by A35R and M1R.

E.Changes in body weight of mice after MPXV infection.

F.Viral Replication in Blood of Mice Infected with MPXV.

G.Viral Hemorrhagia and Pathological Score in the Blood of Mice Infected with MPXV.

H.Lung pathology.
